# The association between physical activity and low back pain: a systematic review and meta-analysis of observational studies

**DOI:** 10.1038/s41598-019-44664-8

**Published:** 2019-06-03

**Authors:** Hosam Alzahrani, Martin Mackey, Emmanuel Stamatakis, Joshua Robert Zadro, Debra Shirley

**Affiliations:** 1 Discipline of Physiotherapy, Faculty of Health Sciences, The University of Sydney, Sydney, 2141 Australia; 2 Department of Physiotherapy, College of Applied Medical Sciences, Taif University, Taif, 21974 Saudi Arabia; 3 Charles Perkins Centre, Prevention Research Collaboration, Sydney School of Public Health, The University of Sydney, Sydney, 2006 Australia; 4 Sydney School of Public Health, Sydney Medical School, The University of Sydney, Sydney, 2050 Australia

**Keywords:** Epidemiology, Risk factors

## Abstract

The aim of this review was to investigate the association between total and domain-specific physical activity (PA) and non-specific low back pain (LBP) in adults. Seven databases were searched for cohort and cross-sectional studies. Pooled estimates of the association of medium and high levels PA and LBP, using the generic inverse-variance method with fixed- and random-effects models were calculated. Twenty-four studies (15 cohort and nine cross-sectional; 95,796 participants) were included. The pooled fully adjusted risk ratios (RR) from cohort studies comparing medium with lowest activity levels were 0.90 (95%CI 0.85 to 0.96) for total PA, and 0.90 (95%CI 0.85 to 0.96) for leisure-time PA (LTPA). The pooled RR comparing highest with lowest activity levels were 1.00 (95%CI 0.92 to 1.08) for total PA, and 1.01 (95%CI 0.93 to 1.10) for LTPA. The pooled fully adjusted odds ratios (OR) from cross-sectional studies comparing medium with lowest activity levels were 0.93 (95%CI 0.65 to 1.32) for total PA, and 0.77 (95%CI 0.62 to 0.96) for LTPA. The pooled OR comparing highest with lowest activity levels were 1.05 (95%CI 0.89 to 1.23) for total PA, and 0.85 (95%CI 0.79 to 0.93) for LTPA. PA seems to be associated with lower prevalence of LBP.

## Introduction

Low back pain (LBP) is a highly prevalent, disabling and costly condition^1,2^. The lifetime prevalence of LBP is reported to be as high as 84%, and the 1-year prevalence is estimated to be up to 65%^3^. Most cases of LBP are classified as non-specific where a specific aetiology has not been determined^4^. Low back pain adversely affects individuals, their families, communities and governments worldwide^5^. Furthermore, the economic burden of LBP is growing due to absenteeism from work, loss of productivity, and cost of treatment^6^.

It is widely recognized that physical activity has numerous health benefits related to physiological and psychological health^7–10^. Physical activity is defined as “any bodily movements produced by skeletal muscles that result in energy expenditure^11^”. Understanding the links between physical activity and LBP will inform future multi-component interventions aimed at LBP prevention. Examining each domain of physical activity separately will enable easier translation of the knowledge generated in observational studies into real-life prevention.

Although it is well known that physical activity can reduce all-cause mortality and risk factors of a wide variety of chronic diseases such as cardiovascular and respiratory diseases, diabetes, obesity and musculoskeletal diseases^8,9^, there are conflicting reports about the associations between physical activity and LBP. For example, one review^12^ failed to find an association between physical activity and LBP, although these results might be explained by differences in the physical activity characteristics across included studies (e.g. type, intensity and duration) and the inclusion of only three studies that examined the association between physical activity and LBP in adults. In addition, the mechanisms of the association between physical activity and LBP remain ambiguous. It has been hypothesized that physical inactivity may lead to reduced muscle strength and flexibility and consequently may result in the spine being susceptible to injuries^13^, although empirical evidence is lacking.

Another related review^14^ suggested that the type and intensity of the physical activity should be considered when measuring the association with LBP. In relation to the domain and level of physical activity, a recent meta-analysis of cohort studies^15^ reported that medium to high level of leisure-time physical activity (LTPA) reduces the risk of developing chronic LBP risk by 11–16%. However, this review did not specify a target population, the outcomes, or statistical approach. For example, it included both adolescents and adults, combined other LBP-related factors such as pain intensity and sick leave in the same analyses, did not always report fully adjusted pooled estimates and failed to explore the influence of different domains of physical activity. A broader understanding of the association between total and domain-specific physical activity (e.g. LTPA, transportation and domestic) and LBP is therefore necessary.

The aim of our systematic review and meta-analysis was to investigate the association between total and domain-specific physical activity and LBP in adults.

## Methods

### Design

This study is a systematic review of published cohort and cross-sectional studies. The protocol detailing the objectives and methods of this systematic review was pre-registered with the International Prospective Register of Systematic Reviews (PROSPERO) (registration number CRD42015027441). This review was conducted in accordance with the Preferred Reporting Items for Systematic reviews and Meta-Analyses (PRISMA) guidelines^16^.

### Identification and selection of studies

The following databases were searched from the earliest records to March 2017: PubMed, Medline, Scopus, CINAHL, EMBASE, SPORTDiscus, Cochrane Library and Web of Science. The search strategy included keywords related to physical activity and LBP. The reference lists of the identified papers and systematic reviews were also checked for additional papers. The full electronic search strategy is provided in Supplementary Table 1.

The information sources were searched independently by two reviewers (HA, JZ). The reviewers independently screened the identified papers for inclusion using the registered protocol and made decisions about inclusion according to the eligibility criteria. Disagreements were resolved by consensus or third reviewer (DS). Initially titles were screened, then abstracts and then full papers.

**Figure 1:**
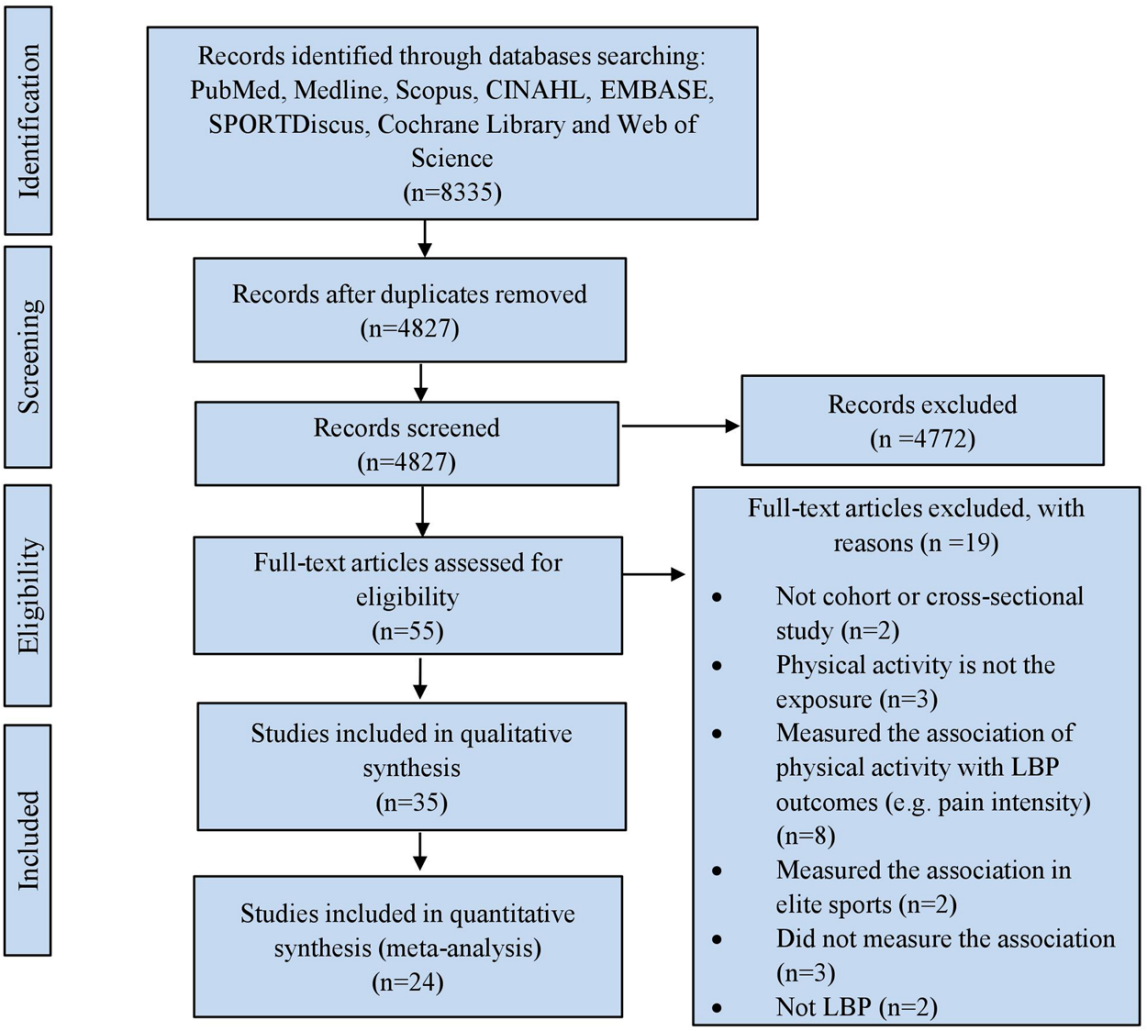
Flow of studies through the review.

A paper was considered potentially relevant and the full text reviewed if, following discussion between the two independent reviewers, it could not be unequivocally excluded on the basis of its title and abstract^16,17^. The full text of all papers not excluded on the basis of title or abstract were screened. The number of articles included and excluded at the different phases was recorded as recommended^18^ and presented in a PRISMA flowchart (Fig. 1).

### Eligibility criteria

The studies were included if they met the following criteria:

- Observational study design (cohort or cross-sectional).
- The study included males and/or females aged 18 years and over with non-specific LBP.
- The study reported estimates of the association between physical activity (exposure) and LBP (outcome).

Studies were excluded if they:

- were experimental or intervention studies;
- used a case-control design;
- were published in languages other than English;
- included participants with LBP due to a specific cause such as serious pathology, fracture, herniated intervertebral disc, neurological compromise, osteoporosis, cancer or other specific causes;
- focused or reported estimates on occupational activity only (e.g. frequent bending/twisting of spine);
- investigated specific professional or elite sports (e.g. soccer players and athlete);
- examined the association between physical activity and LBP-related outcomes (e.g. pain intensity) only.

### Data extraction

Data were extracted independently by three reviewers (HA, DS, JZ) using a data extraction form tailored to the requirements of this systematic review. Disagreements were resolved through discussion between the reviewers. The extracted data included first author, study design, study population, participant characteristics, sample size, prevalence of LBP, physical activity measurements, and main findings. The corresponding author of eligible studies were contacted if potentially relevant data were missing.

The included studies in the quantitative syntheses followed different methods in measuring and classifying physical activity. Therefore, physical activity variables were harmonised to the common unit of metabolic equivalent (MET)-hours/week to facilitate integration of activities differing in intensity and duration accumulated over a week. If physical activity volume per week (MET-hours/week) was not reported, we calculated the MET-hours/week. Intensity weighted volume expressed in MET-hours/week was calculated as MET for each activity multiplied by amount of time spent (frequency × duration) on that activity per week^19^. A standard compendium of MET values for many different activities can be found on the Compendium^19^. However, some studies did not report the intensity, duration or frequency for the measured activity. Therefore, a number of standard a priori rules were followed for assigning the dose in MET-hours/week for all extracted physical activity variables from the included studies, to remove subjectivity from the decision-making process. These rules were reported in previous published reviews^20–22^.

When a study expressed physical activity as a specific activity (e.g. walking, cycling, gardening, etc.) and its duration, we defined the intensity of the activity according to the Compendium: gardening, 5.5 METs; cycling, 7.5 METs; swimming, 6 METs; aerobics, 5.5 METs; jogging, 7.3 METs; brisk walking, 4.3 METs^19^. When a study described physical activity in terms of intensity (rather than describing the actual activity that was performed), then we assigned the following average intensity for each intensity level: light intensity: 3 METs; moderate intensity: 4 METs; moderate to vigorous: 4.5; vigorous intensity: 8 METs^19,21,23^.

We also modified and added some other rules to fit our study. The rules for converting physical activity measures to a standardized MET-hours/week are listed in Supplementary Table 2.

### Assessment of study quality

The included studies were assessed for methodological quality using the Downs and Black checklist^24^ modified from a previous systematic review^25^. More modifications were made to the checklist based on the study designs included in this review (Supplementary Table 3). The assessment was conducted by two reviewers (HA, JZ) independently. Disagreements were resolved by discussion or consultation with a third reviewer. Nineteen criteria were used to assess the methodological quality of cohort studies and 15 for cross-sectional studies. A mid-point score (>50%) was defined as a cut-off to identify high-quality studies^26^. Therefore, cohort studies scoring more than 9 and cross-sectional studies scoring more than 7 were identified as high-quality studies.

### Data synthesis and analysis

The main analysis was conducted to examine the association of total physical activity with LBP. We aimed also to conduct subgroup analyses concerning domain-specific physical activity (LTPA, transportation and domestic). We were able to conduct subgroup analyses concerning LTPA, but not the other domains (transportation and domestic) due to insufficient data. Design-specific meta-analyses were conducted for cohort and cross-sectional studies. If a study presented results for more than one specific-type or domain of physical activity separately (e.g. walking and gardening), all types of physical activity were included in the analysis as independent variables. However, if a study presented the wider spectrum of physical activity as well (e.g. total physical activity, total LTPA), it was the only type selected to avoid duplication. If a study measured the association with different types of LBP, only the initial episode of LBP was included in the analysis.

All the extracted variables of physical activity from included studies were categorized into groups defined around tertiles. Two groups were included in the analyses of total physical activity: (a) ‘medium‘ was between the 33^th^ and 66^th^ percentile (reporting >11.89 and <21 MET-hours/week), and (b) ‘high‘ was greater than or equal to the 66^th^ percentile (reporting ≥21 MET-hours/week). Two groups were included in the analyses of LTPA: (a) ‘medium‘ was between the 33^th^ and 66^th^ percentile (reporting >11.76 and <18.67 MET-hours/week), and (b) ‘high‘ was greater than or equal to the 66^th^ percentile (reporting ≥18.67 MET-hours/week).

The data were pooled and synthesised using the Review Manager (RevMan) program^27^. We computed fully adjusted risk ratio (RR) or odds ratio (OR) comparing the: (1) medium level of physical activity with the lowest level (reference category); and (2) high level of physical activity with the lowest level. In some studies, the reported RR or OR used the highest or middle category of physical activity level rather than the lowest as the reference category; in this case we inverted the reported values by dividing 1 on the RR or OR and the confidence intervals (CIs) (1/RR or 1/OR; 1/upper boundary, 1/lower boundary)^28^.

In this review, RR and OR were used as the common measures of association for the meta-analyses of cohort and cross-sectional studies, respectively. The data were pooled and calculated as the inverse variance weighted mean of the logarithms of the RR or OR with their 95% CI^29^. For cohort studies that measured the association between the variables using different measures such as hazard ratio (HR), we interpreted them as RR estimate^28^. For cross-sectional studies that measured the association using different measures such as RR, we interpreted them as OR estimate^30^. When a study reported the results for different groups separately (e.g. different age groups, or male and female), all groups were included in the meta-analysis because they were independent from each other.

Statistical heterogeneity among studies was assessed using the Higgins *I*^2^ test^31^. A value of *I*^2^ greater than 50% is considered to indicate large heterogeneity^31,32^. In the presence of large heterogeneity, a random effects model was used; otherwise, a fixed effects model was used^29^. The result was considered to be statistically significant when the *P* value was less than 0.05; or if the 95% CI about the RR or OR did not cross 1. Publication bias was assessed using funnel plots and Egger’s test^33^ when more than 10 studies were included in a meta-analysis^34^.

### Sensitivity analysis

We examined the robustness of the results by including only high-quality studies (based on a cut-off of >50% from the modified Downs and Black checklist). However, because all the included studies were classed as high quality (scoring >50%), we increased the cut-off point to >70%. Cohort studies scoring more than 13 out of 19 and cross-sectional studies scoring more than 10 out of 15 were included in these sensitivity analyses. The effect of the including of high-quality studies (scoring >50%) on the pooled results was then examined by repeating the analysis using only very high-quality studies (scoring >70%).

## Results

### Search results

The searches retrieved 8335 studies. After removing duplicates, 4827 studies remained. Screening these studies by titles and abstracts indicated 55 studies were eligible for assessment by full paper. Of these, 35 studies^35–69^ (106,776 participants; 49.8% female) fulfilled the inclusion criteria for further analysis (Supplementary Table 4), and 24 of these studies were included in the quantitative syntheses (Supplementary Table 5). Eleven included studies were not included in the quantitative syntheses because they did not consider adjustment for potential confounding factors^40,42,46,47,53,54,57,58,60,68^ or due to lack of clarity of results^63^.

The 24 studies consisted of 15 cohort studies^36,38,39,41,44,52,55,56,59,61,62,64–67^ and 9 cross-sectional studies^35,37,43,45,48–51,69^. The flow of studies through the review is depicted in Fig. 1.

### Study characteristics

The total sample size of the 35 included studies comprised subjects (range, 68–32417), with an age range of 18–100 years, with approximately equal balance between females and males. Most studies (n = 33) recruited both male and female participants, and two studies^51,64^ recruited only male participants. The characteristics of the included studies are described in Supplementary Table 4.

Twenty-four studies comprising 95,796 participants were included in the quantitative syntheses (meta-analyses). Most of these studies were adjusted for age (n = 20), gender (n = 19), and smoking (n = 14). Other potential confounding factors were considered in less than 50% of studies and included body mass index (BMI), education, ethnicity, income, stress, anxiety, depression, fear of pain, alcohol consumption, occupation, self-rated health, overweight, obesity, sleep quality, chronic disease history, cardiorespiratory, hypercholesterolemia, diabetes, hypertension, pain management, medication use, consultation, musculoskeletal symptoms or injuries, surgery, disability, total hip osteoporosis status, residential area, nutritional level, fitness, father’s occupation, whole body vibration, occupational activities, and other types of activities) (Supplementary Table 5).

### Quality assessment

Nineteen out of 20 cohort studies^36,38,39,41,42,44,46,52,55,56,59,61–68^ and 13 out of 15 cross-sectional studies^35,37,40,43,45,47–51,53,54,69^ were rated as high quality using the modified Downs and Black checklist criteria (Supplementary Table 6). The mean score of the quality assessment was 14.2 out of 19 for the cohort studies (range, 9–17) and 10 out of 14 for the cross-sectional studies (range, 5–14). All the included studies that were eligible to be included in the analyses were assessed to be of high quality.

### Physical activity measurements

The majority of the included studies (n = 33) used self-administered questionnaires to measure physical activity and only two studies^63,65^ used objective measurements of physical activity (Digi-walker Pedometer CW700s and Actigraph accelerometer). Of the 24 studies included in the meta-analyses, seven studies assessed total physical activity^43,48–50,65–67^ and nine assessed total LTPA or recreational physical activity^36,37,43–45,52,59,62,69^. Different types and intensities of physical activity were assessed in 11 studies^35,38,41,43,45,50,51,55,61,62,64^ (Supplementary Table 7).

### The association between total physical activity and low back pain

#### Medium level versus low level total physical activity

**Figure 2:**
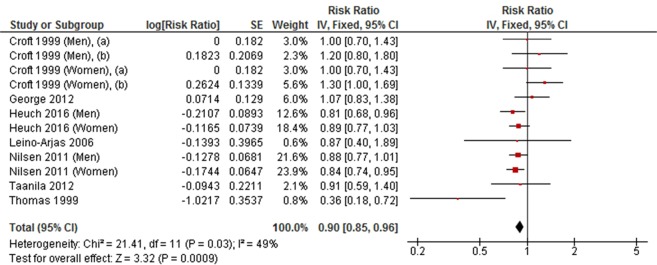
Forest plot of cohort studies investigating the association between medium level versus low level physical activity and low back pain.

**Figure 3:**
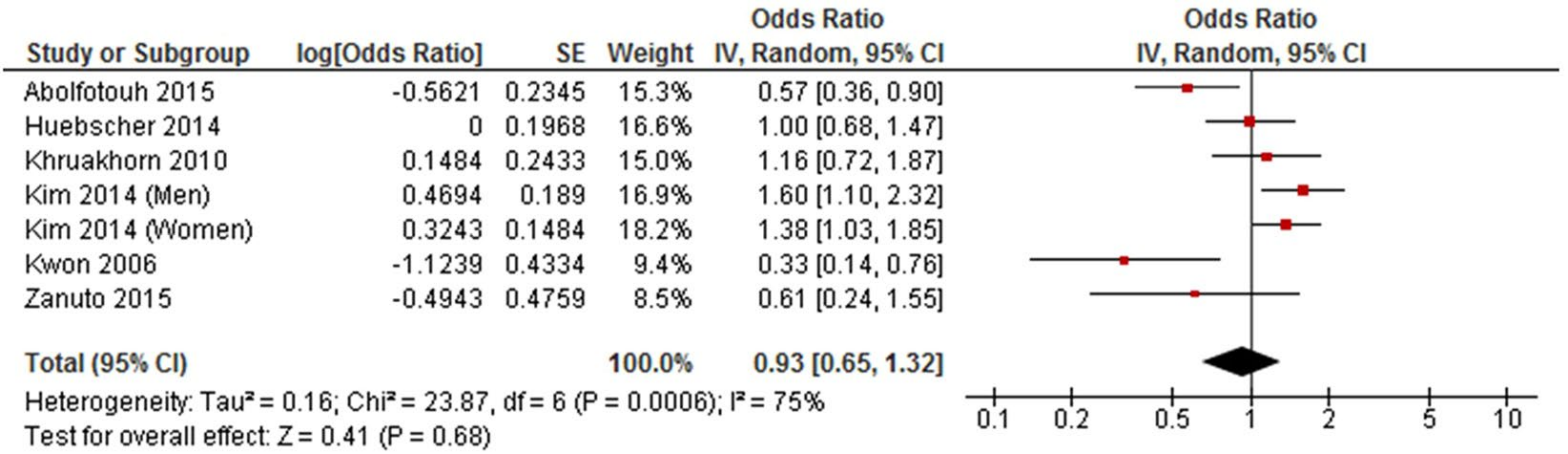
Forest plot of cross-sectional studies investigating the association between medium level versus low level physical activity and low back pain.

Medium level physical activity was significantly associated with a decreased risk of developing LBP in a meta-analysis of seven cohort studies (RR = 0.90, 95% CI 0.85 to 0.96, *P* = 0.0009, *I*^2^ = 49%) (Fig. 2), but was not associated with LBP in a meta-analysis of six cross-sectional studies (OR = 0.93, 95% CI 0.65 to 1.32, *P* = 0.68, *I*^2^ = 75%) (Fig. 3).

#### High level versus low level total physical activity

**Figure 4:**
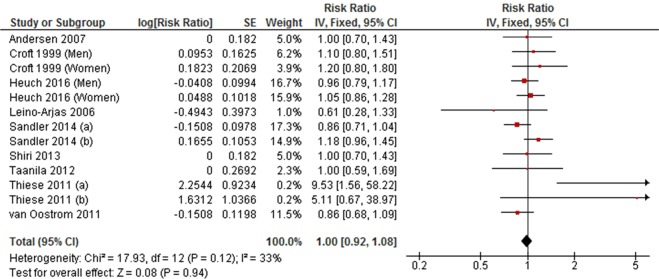
Forest plot of cohort studies investigating the association between high level versus low level physical activity and low back pain.

**Figure 5:**
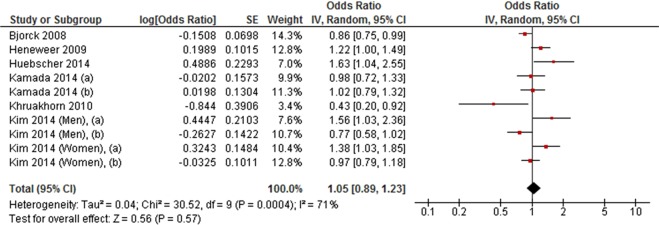
Forest plot of cross-sectional studies investigating the association between high level versus low level physical activity and low back pain.

High level physical activity was not associated with LBP in a meta-analysis of nine cohort studies (RR = 1.00, 95% CI 0.92 to 1.08, *P* = 0.94; *I*^2^ = 33%) (Fig. 4). This finding was consistent with a meta-analysis of six cross-sectional studies (OR = 1.05, 95% CI 0.89 to 1.23, *P* = 0.57; *I*^2^ = 71%) (Fig. 5).

### The association between leisure-time physical activity and low back pain

#### Medium level versus low level leisure-time physical activity

**Figure 6:**
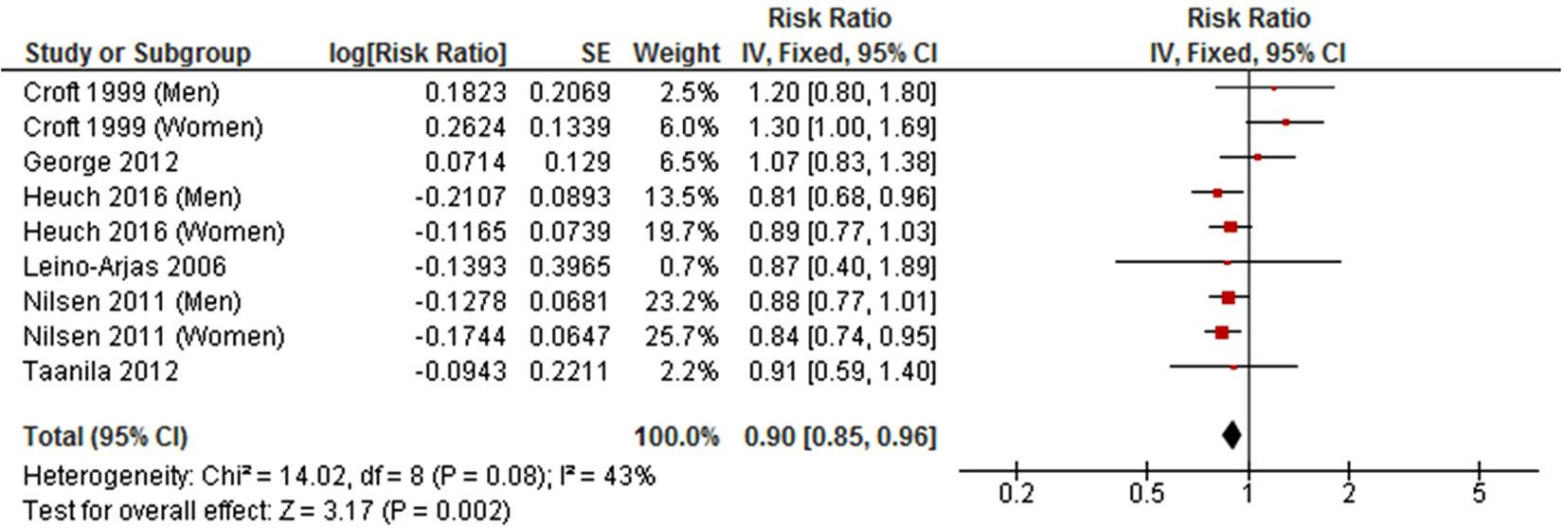
Forest plot of cohort studies investigating the association between medium level versus low level leisure-time physical activity and low back pain.

**Figure 7:**
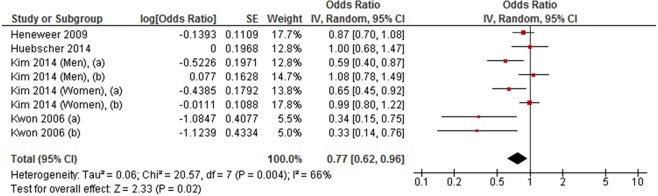
Forest plot of cross-sectional studies investigating the association between medium level versus low level leisure-time physical activity and low back pain.

A meta-analysis of six cohort studies showed that medium level LTPA was inversely associated with LBP (RR = 0.90, 95% CI 0.85 to 0.96, *P* < 0.002; *I*^2^ = 43%) (Fig. 6). A similar pattern of association was also observed in a meta-analysis of four cross-sectional studies between medium level LTPA and LBP (OR = 0.77, 95% CI 0.62 to 0.96, *P* = 0.02; *I*^2^ = 66%) (Fig. 7).

#### High level versus low level leisure-time physical activity

**Figure 8:**
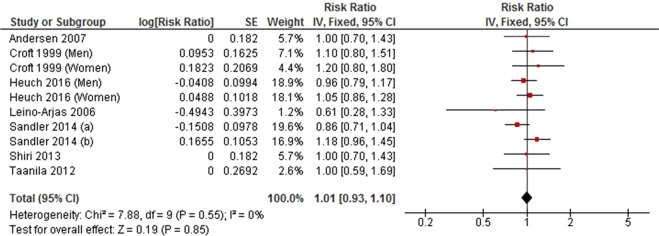
Forest plot of cohort studies investigating the association between high level versus low level leisure-time physical activity and low back pain.

**Figure 9:**
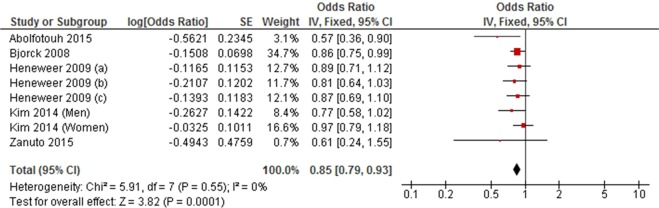
Forest plot of cross-sectional studies investigating the association between high level versus low level leisure-time physical activity and low back pain.

Seven cohort studies and five cross-sectional studies examined the association between high level LTPA and LBP. The meta-analyses of cohort studies did not find an association between high level LTPA and LBP (RR = 1.01, 95% CI 0.93 to 1.10, *P* = 0.85; *I*^2^ = 0%) (Fig. 8). This finding was inconsistent with the result of the meta-analysis of cross-sectionals studies (OR = 0.85, 95% CI 0.79 to 0.93, *P* = 0.0001; *I*^2^ = 0%) (Fig. 9).

### Sensitivity analysis

The sensitivity analyses restricted to very high-quality studies (cohort and cross-sectional studies scoring >13 and >10, respectively, using the modified Downs and Black checklist criteria) did not change the results significantly.

## Discussion

We found an inverse association between physical activity and LBP, but we found no evidence of dose-response.

The pooled results from cohort studies demonstrated that people engaged in medium level physical activity have a 10% lower risk of LBP, compared to those engaged in low level physical activity. When only cross-sectional studies were considered, this association disappeared, although the results suggested a 7% decreased odds of LBP (non-significant). However, a large heterogeneity was observed across cross-sectional studies (*I*^2^ = 75%), reflected in the random effects estimate and the wide CIs (CI 0.65 to 1.32, which include the null effect). A potential explanation for this heterogeneity is the inclusion of a low number of studies and the variability in measures of physical activity among these studies. Moreover, the current analyses found no association between high level physical activity and LBP, compared to low level physical activity. This finding was stable across both cohort and cross-sectional studies.

Our results were inconsistent with the review conducted by Sitthipornvorakul, *et al*.^12^ which found conflicting results for the association between physical activity and LBP; however, that review included only three cross-sectional studies, published up to 2009 and reported the association of physical activity with LBP in adults. Moreover, in contrast to that review, our study explored the association between physical activity and LBP quantitatively, and included more studies and larger sample size.

The subgroup analyses of pooled results from cohort studies showed that people engaged in medium level LTPA have a 10% lower risk of LBP, whilst there was no association with high level LTPA. The absence of an association between high level LTPA and LBP might be due to including different types and durations of LTPA which may have resulted in misclassification, and then underestimation of the effect of high level LTPA. When only cross-sectional studies were included, we found a 23% and 15% odds reduction in LBP for medium and high levels LTPA, respectively. These results are consistent with the results of the review conducted by Shiri and Falah-Hassani^15^ which pooled results from cohort studies of LTPA and showed 14% and 16% decreased risk of chronic LBP for medium and high levels, respectively. Although there are some differences between the two studies detailed above in terms of classification and levels of physical activity, both studies appear to agree about the importance of medium to high levels LTPA in decreasing the risk of LBP. Therefore, this strengthens the evidence base supporting the protective role that physical activity has on the development of LBP.

The results of the meta-analyses did not change when we restricted the analyses to very high-quality studies suggesting the results were robust against bias. The following potential sources of bias were identified: nine cohort studies (out of 15) failed to describe the characteristics of patients who were lost to follow-up and therefore it is not known whether their inclusion would affect the results, and eleven cohort studies did not use a reliable and/or valid measure of physical activity outcome which may impact the internal validity. Additionally, seven cohort studies and six cross-sectional studies (out of 9) failed to report the proportion of participants who agreed to participate in the study and whether they were representative of the source population which may lead to a possible threat to the external validity (i.e. generalisability of the study results).

To the best of our knowledge, our study is the first to quantitatively investigate the association between physical activity and LBP by expressing physical activity in MET-hours/week. This likely explains any discrepancies between the findings of this review with previous reviews, and highlights the importance of determining the frequency, duration, intensity and type of physical activity that has the greatest protective effect against the development of LBP. A cost-benefit analysis of physical activity could not be conducted as none of the included studies investigated cost-benefit as a mediating factor for the relationship between physical activity and LBP. However, if engaging in medium level physical activity reduces the risk of LBP, it may also reduce the cost of health care associated with LBP. Therefore, future studies including a cost-benefit analyses of physical activity are needed.

Occupational activity was excluded in this review due to the large variation in methods of measuring each activity such as frequent lifting and bending/twisting of spine, and the difficulty to accurately quantify the METs and the time spent on that activities. Therefore, it is difficult to classify these activities and include them in the analyses with other types of non-occupational physical activity. However, participation in occupational activities including frequent lifting and physically demanding workload was found to be a medium to strong risk factor for LBP^14^. Therefore, future research should adjust for both occupational and non-occupational physical activities which reflect the daily exposure of individuals to all types of activities. In our study, of the 24 studies included in our analyses, only a few studies adjusted for occupational activities and for other types of non-occupation physical activity.

The strengths of this systematic review include: (1) comprehensive literature search strategy including eight databases; (2) all the included studies were assessed to be of acceptable quality; (3) we included fully adjusted models from each study in the analyses to reduce the possibility of confounding; and (4) it confirmed the main findings through a sensitivity analyses of only very high-quality studies.

Several limitations should also be considered when interpreting the results of this review. First, most included studies used self-administered questionnaires in measuring physical activity that likely produce recall bias and overestimation^70^. Second, measurements and classifications of physical activity in terms of frequency, intensity and duration differed across included studies which may lead to misclassification of physical activity levels. Third, the adjusted models varied across the included studies, and it was not possible to adjust for some potential risk factors, including occupational and non-occupational physical activity types.

In conclusion, the results of our study provide evidence suggesting there is an inverse association between physical activity and LBP. Medium activity level was associated with lower prevalence of LBP. These findings may have implications for including moderate doses of physical activity in the management and prevention of LBP in clinical practice.

## Supplementary information

SUPPLEMENTARY INFORMATION
