# Author Correction: The association between physical activity and low back pain: a systematic review and meta-analysis of observational studies

**DOI:** 10.1038/s41598-020-62512-y

**Published:** 2020-04-01

**Authors:** Hosam Alzahrani, Martin Mackey, Emmanuel Stamatakis, Joshua Robert Zadro, Debra Shirley

**Affiliations:** 1 Discipline of Physiotherapy, Faculty of Health Sciences, The University of Sydney, Sydney, 2141 Australia; 2 Department of Physiotherapy, College of Applied Medical Sciences, Taif University, Taif, 21974 Saudi Arabia; 3 Charles Perkins Centre, Prevention Research Collaboration, Sydney School of Public Health, The University of Sydney, Sydney, 2006 Australia; 4 Sydney School of Public Health, Sydney Medical School, The University of Sydney, Sydney, 2050 Australia

Correction to: *Scientific Reports* 10.1038/s41598-019-44664-8, published online 03 June 2019

The original version of this Article omitted an affiliation for Hosam Alzahrani. The correct affiliations are listed below:

Discipline of Physiotherapy, Faculty of Health Sciences, The University of Sydney, Sydney, Australia.

Department of Physiotherapy, College of Applied Medical Sciences, Taif University, Taif, Saudi Arabia.

This has now been corrected in the HTML and PDF versions of the Article, and in the accompanying Supplementary Information file.

