# Supplementary material for: The association between physical activity and low back pain: a systematic review and meta-analysis of observational studies
Source: Sci Rep. 2019 Jun 3;9:8244. doi: 10.1038/s41598-019-44664-8 (PMC6547713; doi:10.1038/s41598-019-44664-8)
Supplement: Supplementary file 1 — SUPPLEMENTARY INFORMATION [file 41598_2019_44664_MOESM1_ESM.docx]

**Supplementary Information**

**The association between physical activity and low back pain: a systematic review and meta-analysis of observational studies**

1. Hosam Alzahrani, MPT, Discipline of Physiotherapy, Faculty of Health Sciences, The University of Sydney, Sydney, Australia; Department of Physiotherapy, College of Applied Medical Sciences, Taif University, Taif, Saudi Arabia. [halz2656@uni.sydney.edu.au](mailto:halz2656@uni.sydney.edu.au)

2. Martin Mackey, PhD, Discipline of Physiotherapy, Faculty of Health Sciences, The University of Sydney, Sydney, Australia. [martin.mackey@sydney.edu.au](mailto:martin.mackey@sydney.edu.au)

3. Emmanuel Stamatakis, PhD, Charles Perkins Centre, Prevention Research Collaboration, Sydney School of Public Health, The University of Sydney, Sydney, Australia. [emmanuel.stamatakis@sydney.edu.au](mailto:emmanuel.stamatakis@sydney.edu.au)

4. Joshua Robert Zadro, BAppSc (Phty) (Hons1), Sydney School of Public Health, Sydney Medical School, The University of Sydney, Sydney, Australia. [jzad3326@uni.sydney.edu.au](mailto:jzad3326@uni.sydney.edu.au)

5. Debra Shirley, PhD, Discipline of Physiotherapy, Faculty of Health Sciences, The University of Sydney, Sydney, Australia. [debra.shirley@sydney.edu.a](mailto:debra.shirley@sydney.edu.a)

**Corresponding author:**

| Name | Hosam Alzahrani |
| --- | --- |
| ORCID | <https://orcid.org/0000-0002-4383-115X> |
| Department | Discipline of Physiotherapy |
| Institution | Faculty of Health Sciences |
| Country | Australia |
| Tel | +61 2 9351 9546 |
| Mob | +61 450101435 |
| Email | [halz2656@uni.sydney.edu.au](mailto:halz2656@uni.sydney.edu.au) |
| Postal address | The University of Sydney  Room S223, Building S \| 75 East St, Lidcombe, NSW 2141 |

**Supplementary Information**

**Supplementary List (In order of appearance in manuscript text):**

**Supplementary Table 1.** Search strategy (Pubmed).

**Supplementary Table 2.** Standard rules for converting physical activity measures to standardized metric of MET-hours/week.

**Supplementary Table 3.** Modified Downs and Black quality checklist to assess measurement of physical activity in low back pain populations.

**Supplementary Table 4.** Characteristics of included studies.

**Supplementary Table 5.** Studies included in the meta-analyses.

**Supplementary Table 6.** Methodological quality assessment of included studies.

**Supplementary Table 7.** Comparison groups included in the meta-analyses.

**Supplementary Table 1. Search strategy (Pubmed)**

| **Search** | **Query** | **Items found** |
| --- | --- | --- |
| [**#1**](https://www.ncbi.nlm.nih.gov/pubmed) | **Search (((((("back pain"[Title/Abstract] OR "low back pain"[Title/Abstract]) OR "lower back pain"[Title/Abstract]) OR "lumbar pain"[Title/Abstract]) OR "spinal pain"[Title/Abstract]))** | [**40904**](https://www.ncbi.nlm.nih.gov/pubmed/?cmd=HistorySearch&querykey=1) |
| [**#2**](https://www.ncbi.nlm.nih.gov/pubmed) | **Search (((((((("physical activity"[All Fields] OR "physical activities"[All Fields]) OR sedentary[All Fields]) OR ("leisure activities"[MeSH Terms] OR ("leisure"[All Fields] AND "activities"[All Fields]) OR "leisure activities"[All Fields] OR "leisure"[All Fields])) OR (("recreation"[MeSH Terms] OR "recreation"[All Fields]) OR recreation'[All Fields] OR recreation's[All Fields] OR recreationa[All Fields] OR recreational[All Fields] OR recreational'[All Fields] OR recreationale[All Fields] OR recreationalists[All Fields] OR recreationally[All Fields] OR recreationand[All Fields] OR recreationers[All Fields] OR recreationism[All Fields] OR recreationist[All Fields] OR recreationist's[All Fields] OR recreationists[All Fields] OR recreationists'[All Fields] OR ("recreation"[MeSH Terms] OR "recreation"[All Fields] OR "recreations"[All Fields]) OR recreationsal[All Fields] OR recreationsouthwest[All Fields] OR recreationtourism[All Fields] OR recreationuniversity[All Fields])) OR (("walking"[MeSH Terms] OR "walking"[All Fields] OR "walk"[All Fields]) OR walk'[All Fields] OR walk''[All Fields] OR walk'n[All Fields] OR walk's[All Fields] OR walk0060[All Fields] OR walk11[All Fields] OR walk2[All Fields] OR walk2bactive[All Fields] OR walk2iron[All Fields] OR walk3m[All Fields] OR walk4life[All Fields] OR walka[All Fields] OR walkabilit[All Fields] OR walkability[All Fields] OR walkability'[All Fields] OR walkability's[All Fields] OR walkable[All Fields] OR walkable'[All Fields] OR walkablilty[All Fields] OR walkabout[All Fields] OR walkabout'[All Fields] OR walkabouts[All Fields] OR walkadoo[All Fields] OR walkaide[All Fields] OR walkaide2[All Fields] OR walkaloner200[All Fields] OR walkanis[All Fields] OR walkar[All Fields] OR walkarcinosarcoma[All Fields] OR walkaround[All Fields] OR walkaround'[All Fields] OR walkarounds[All Fields] OR walkate[All Fields] OR walkathon[All Fields] OR walkathons[All Fields] OR walkato[All Fields] OR walkaway[All Fields] OR walkaway'[All Fields] OR walkaway40[All Fields] OR walkaway40plus[All Fields] OR walkawaytrade[All Fields] OR walkay[All Fields] OR walkaz[All Fields] OR walkback[All Fields] OR walkbility[All Fields] OR walkbot[All Fields] OR walkbristol[All Fields] OR walkden[All Fields] OR walkdenandrew[All Fields] OR walkdist[All Fields] OR walkdown[All Fields] OR walkdr4[All Fields] OR walke[All Fields] OR walke009[All Fields] OR walke01[All Fields] OR walke082[All Fields] OR walke284[All Fields] OR walked[All Fields] OR walked'[All Fields] OR walkeded[All Fields] OR walkeden[All Fields] OR walkefield[All Fields] OR walkefii[All Fields] OR walkei[All Fields] OR walkejb[All Fields] OR walkem[All Fields] OR walkembach[All Fields] OR walkemei[All Fields] OR walkemeier[All Fields] OR walkemeyer[All Fields] OR walken[All Fields] OR walkenaer[All Fields] OR walkenbach[All Fields] OR walkenfeld[All Fields] OR walkenfort[All Fields] OR walkenhorst[All Fields] OR walkenshaw[All Fields] OR walkenstein[All Fields] OR walkenstrom[All Fields] OR ("walkers"[MeSH Terms] OR "walkers"[All Fields] OR "walker"[All Fields]) OR walker'[All Fields] OR walker's[All Fields] OR walker00[All Fields] OR walker03[All Fields] OR walker06[All Fields] OR walker09[All Fields] OR walker1[All Fields] OR walker11[All Fields] OR walker2[All Fields] OR walker2004[All Fields] OR walker256[All Fields] OR walker3[All Fields] OR walker36[All Fields] OR walker4[All Fields] OR walker45[All Fields] OR walker456[All Fields] OR walker55[All Fields] OR walker6[All Fields] OR walker7[All Fields] OR walker80[All Fields] OR walker88[All Fields] OR walker9[All Fields] OR walkera[All Fields] OR walkera1[All Fields] OR walkerae[All Fields] OR walkeraj[All Fields] OR walkeram[All Fields] OR walkerana[All Fields] OR walkerana'[All Fields] OR walkerassisted[All Fields] OR walkerb[All Fields] OR walkerbj[All Fields] OR walkerbr[All Fields] OR walkerc[All Fields] OR walkerc1[All Fields] OR walkercarcinom[All Fields] OR walkerch[All Fields] OR walkercompany[All Fields] OR walkerd[All Fields] OR walkerd4[All Fields] OR walkerdeena[All Fields] OR walkerdf[All Fields] OR walkerdr1986[All Fields] OR walkere[All Fields] OR walkere1[All Fields] OR walkereconomics[All Fields] OR walkerella[All Fields] OR walkerella'[All Fields] OR walkeres[All Fields] OR walkerette[All Fields] OR walkerfield[All Fields] OR walkerg[All Fields] OR walkerg1[All Fields] OR walkergate[All Fields] OR walkergatepark[All Fields] OR walkergi68[All Fields] OR walkergky[All Fields] OR walkergp[All Fields] OR walkerh[All Fields] OR walkerhao[All Fields] OR walkeri[All Fields] OR walkeri'[All Fields] OR walkeriana[All Fields] OR walkerianus[All Fields] OR walkerii[All Fields] OR walkerina[All Fields] OR walkerit[All Fields] OR walkerj[All Fields] OR walkerj3[All Fields] OR walkerja[All Fields] OR walkerjf[All Fields] OR walkerjk[All Fields] OR walkerjm[All Fields] OR walkerjr[All Fields] OR walkerk1[All Fields] OR walkerka[All Fields] OR walkerkarzinom[All Fields] OR walkerkarzinoms[All Fields] OR walkerkd[All Fields] OR walkerkr[All Fields] OR walkerkw[All Fields] OR walkerl[All Fields] OR walkerley[All Fields] OR walkerlin[All Fields] OR walkerlr[All Fields] OR walkerls[All Fields] OR walkerm[All Fields] OR walkerma[All Fields] OR walkermd[All Fields] OR walkermp[All Fields] OR walkern[All Fields] OR walkerov[All Fields] OR walkerov'ym[All Fields] OR walkerova[All Fields] OR walkerovho[All Fields] OR walkerp[All Fields] OR walkerparking[All Fields] OR walkerpd[All Fields] OR walkerpeach[All Fields] OR walkerr[All Fields] OR walkerri[All Fields] OR walkerrj[All Fields] OR walkerrl[All Fields] OR walkerro[All Fields] OR walkerronald[All Fields] OR ("walkers"[MeSH Terms] OR "walkers"[All Fields]) OR walkers'[All Fields] OR walkersamuel[All Fields] OR walkersh[All Fields] OR walkershields[All Fields] OR walkersuse[All Fields] OR walkersville[All Fields] OR walkert[All Fields] OR walkerton[All Fields] OR walkertrade[All Fields] OR walkertumor[All Fields] OR walkerul[All Fields] OR walkeruv[All Fields] OR walkerville[All Fields] OR walkervk[All Fields] OR walkerw[All Fields] OR walkerworld[All Fields] OR walkerzhouhl[All Fields] OR walkes[All Fields] OR walkesar[All Fields] OR walkeshwar[All Fields] OR walkette[All Fields] OR walkey[All Fields] OR walkf[All Fields] OR walkfr[All Fields] OR walkfree[All Fields] OR walkg[All Fields] OR walkgyk[All Fields] OR walkhatib[All Fields] OR walkhoff[All Fields] OR walkhoff's[All Fields] OR walkhoffs[All Fields] OR walkhoffschen[All Fields] OR walkhom[All Fields] OR walkhueb[All Fields] OR walki[All Fields] OR walkie[All Fields] OR walkies[All Fields] OR walkiewi[All Fields] OR walkiewicz[All Fields] OR walkimar[All Fields] OR walkin[All Fields] OR walkin'[All Fields] OR walkinehaw[All Fields] OR walkinf[All Fields] OR ("walking"[MeSH Terms] OR "walking"[All Fields]) OR walking'[All Fields] OR walking's[All Fields] OR walkingcondition[All Fields] OR walkinghrt[All Fields] OR walkingmay[All Fields] OR walkingon[All Fields] OR walkings[All Fields] OR walkingshaw[All Fields] OR walkingspeed[All Fields] OR walkingspree[All Fields] OR walkingstick[All Fields] OR walkingstick's[All Fields] OR walkingsticks[All Fields] OR walkingtime[All Fields] OR walkington[All Fields] OR walkins[All Fields] OR walkinsahaw[All Fields] OR walkinsense[All Fields] OR walkinshaw[All Fields] OR walkinsl[All Fields] OR walkinstown[All Fields] OR walkinton[All Fields] OR walkiria[All Fields] OR walkiria589[All Fields] OR walkit[All Fields] OR walkiw[All Fields] OR walkjimi[All Fields] OR walklab[All Fields] OR walkland[All Fields] OR walklate[All Fields] OR walklederhulse[All Fields] OR walklength[All Fields] OR walkler[All Fields] OR walklet[All Fields] OR walklett[All Fields] OR walkley[All Fields] OR walklike[All Fields] OR walklin[All Fields] OR walklin's[All Fields] OR walkling[All Fields] OR walklink[All Fields] OR walkly[All Fields] OR walkman[All Fields] OR walkman'[All Fields] OR walkmann[All Fields] OR walkmans[All Fields] OR walkmat[All Fields] OR walkmate[All Fields] OR walkmech[All Fields] OR walkmed[All Fields] OR walkmen[All Fields] OR walkmore[All Fields] OR walkmr[All Fields] OR walkmuhlenstrasse[All Fields] OR walkmycin[All Fields] OR walkner[All Fields] OR walknet[All Fields] OR walknowska[All Fields] OR walknt[All Fields] OR walko[All Fields] OR walkoff[All Fields] OR ("Walkolime"[Supplementary Concept] OR "Walkolime"[All Fields] OR "walkolime"[All Fields]) OR walkom[All Fields] OR walkonowska[All Fields] OR walkony[All Fields] OR walkosz[All Fields] OR walkoththamalli[All Fields] OR walkotte[All Fields] OR walkotten[All Fields] OR walkout[All Fields] OR walkouts[All Fields] OR walkov[All Fields] OR walkova[All Fields] OR walkover[All Fields] OR walkovers[All Fields] OR walkovich[All Fields] OR walkow[All Fields] OR walkowa[All Fields] OR walkowiac[All Fields] OR walkowiak[All Fields] OR walkowicz[All Fields] OR walkowitch[All Fields] OR walkowitz[All Fields] OR walkowska[All Fields] OR walkowski[All Fields] OR walkowsky[All Fields] OR walkpath[All Fields] OR walkpp[All Fields] OR walkq[All Fields] OR walkr[All Fields] OR walkrankenhaus[All Fields] OR walkre[All Fields] OR walkria[All Fields] OR walkringen[All Fields] OR walkrk[All Fields] OR walkround[All Fields] OR walkrounds[All Fields] OR walkrounds'[All Fields] OR walkroundstrade[All Fields] OR walkroundtm[All Fields] OR walks[All Fields] OR walks'[All Fields] OR walks4work[All Fields] OR walksacramento[All Fields] OR walksafe[All Fields] OR walksat[All Fields] OR walkscape[All Fields] OR walkscore[All Fields] OR walkshan[All Fields] OR walkshed[All Fields] OR walkshed'[All Fields] OR walksheds[All Fields] OR walkshop[All Fields] OR walkshop'[All Fields] OR walkshops[All Fields] OR walksman[All Fields] OR walkspn[All Fields] OR walkstation[All Fields] OR walkstations[All Fields] OR walksun[All Fields] OR walksweet[All Fields] OR walkt[All Fields] OR walktest[All Fields] OR walkthough[All Fields] OR walkthrough[All Fields] OR walkthrough'[All Fields] OR walkthroughs[All Fields] OR walktime[All Fields] OR walktrainer[All Fields] OR walktrap[All Fields] OR walkty[All Fields] OR walkuba[All Fields] OR walkup[All Fields] OR walkup's[All Fields] OR walkush[All Fields] OR walkuska[All Fields] OR walkuski[All Fields] OR walkusz[All Fields] OR walkvo2[All Fields] OR walkway[All Fields] OR walkway'[All Fields] OR walkway's[All Fields] OR walkways[All Fields] OR walkwell[All Fields] OR walkwithfes[All Fields] OR walkwithmeerkats[All Fields] OR walkwitz[All Fields] OR walkx001[All Fields] OR walkyoto[All Fields] OR walkyria[All Fields] OR walkyrie[All Fields] OR walkzak[All Fields])) OR ("exercise"[MeSH Terms] OR "exercise"[All Fields])) OR "active transportation"[All Fields])))** | [**651524**](https://www.ncbi.nlm.nih.gov/pubmed/?cmd=HistorySearch&querykey=2) |
| [**#3**](https://www.ncbi.nlm.nih.gov/pubmed) | **Search (((associat[All Fields] OR associata[All Fields] OR associata'[All Fields] OR associatat[All Fields] OR associatation[All Fields] OR associatd[All Fields] OR associatdd[All Fields] OR associate[All Fields] OR associate'[All Fields] OR associate'd[All Fields] OR associate'healthy'[All Fields] OR associate's[All Fields] OR associateaarhus[All Fields] OR associateacd[All Fields] OR associated[All Fields] OR associated'[All Fields] OR associated'with[All Fields] OR associated1[All Fields] OR associated101[All Fields] OR associated1a[All Fields] OR associated3[All Fields] OR associated9[All Fields] OR associatedantigen[All Fields] OR associatedassociated[All Fields] OR associatedboth[All Fields] OR associatedbreast[All Fields] OR associatedc[All Fields] OR associatedcarcinogenesis[All Fields] OR associatedcomorbidities[All Fields] OR associatedcompeting[All Fields] OR associatedcomplications[All Fields] OR associateddrosophila[All Fields] OR associatedepartment[All Fields] OR associatedeyecare[All Fields] OR associatedfactors[All Fields] OR associatedfindings[All Fields] OR associatedgastrointestinal[All Fields] OR associatedgenes[All Fields] OR associatedi[All Fields] OR associatedinfections[All Fields] OR associatedinflammation[All Fields] OR associatedivision[All Fields] OR associatedly[All Fields] OR associatedmalformations[All Fields] OR associatedmetabolic[All Fields] OR associatedmutations[All Fields] OR associatedneurodegeneration[All Fields] OR associatedpathologies[All Fields] OR associatedphospholipase[All Fields] OR associatedpollutants[All Fields] OR associatedprotein[All Fields] OR associatedproteins[All Fields] OR associatedretinalconsultants[All Fields] OR associateds[All Fields] OR associatedsymptoms[All Fields] OR associatedvirus[All Fields] OR associatedw[All Fields] OR associatedwith[All Fields] OR associatedwitha[All Fields] OR associatedwithlack[All Fields] OR associatedwithworse[All Fields] OR associatee[All Fields] OR associateed[All Fields] OR associatees[All Fields] OR associateexoneura[All Fields] OR associatefamily[All Fields] OR associateguttmacher[All Fields] OR associateion[All Fields] OR associately[All Fields] OR associatent[All Fields] OR associateon[All Fields] OR associatepityogenes[All Fields] OR associateprofessor[All Fields] OR associaterd[All Fields] OR associates[All Fields] OR associates'[All Fields] OR associates19[All Fields] OR associates1915[All Fields] OR associates195[All Fields] OR associates77[All Fields] OR associatesbaltimore[All Fields] OR associatesboardman[All Fields] OR associatesboston[All Fields] OR associatesbrookline[All Fields] OR associatescorner[All Fields] OR associatesdarien[All Fields] OR associatesdouble[All Fields] OR associateselk[All Fields] OR associatesgreenbelt[All Fields] OR associateship[All Fields] OR associateships[All Fields] OR associateshouston[All Fields] OR associatesinc[All Fields] OR associatesjohnson[All Fields] OR associateskelowna[All Fields] OR associatesllc[All Fields] OR associatesmurfreesboro[All Fields] OR associatesnew[All Fields] OR associatess[All Fields] OR associatesto[All Fields] OR associatesunder[All Fields] OR associateswinter[All Fields] OR associatet[All Fields] OR associatetd[All Fields] OR associateted[All Fields] OR associateuniversity[All Fields] OR associatevicechancellor[All Fields] OR associatexs[All Fields] OR associathed[All Fields] OR associathfed[All Fields] OR associati[All Fields] OR associatian[All Fields] OR associatiated[All Fields] OR associatiates[All Fields] OR associatical[All Fields] OR associatie[All Fields] OR associatied[All Fields] OR associaties[All Fields] OR associatiestudies[All Fields] OR associatietest[All Fields] OR associatieve[All Fields] OR associatif[All Fields] OR associatifs[All Fields] OR associatin[All Fields] OR associating[All Fields] OR associating'[All Fields] OR associatinge[All Fields] OR associatio[All Fields] OR associatioa[All Fields] OR associatiociation[All Fields] OR associatioin[All Fields] OR ("association"[MeSH Terms] OR "association"[All Fields]) OR association'[All Fields] OR association's[All Fields] OR association'with[All Fields] OR association10[All Fields] OR association1970[All Fields] OR association299[All Fields] OR association34[All Fields] OR association5[All Fields] OR association600[All Fields] OR association92[All Fields] OR association93[All Fields] OR association99[All Fields] OR associationachieved[All Fields] OR associational[All Fields] OR associational'[All Fields] OR associationalism[All Fields] OR associationaly[All Fields] OR associationand[All Fields] OR associationb[All Fields] OR associationberlin[All Fields] OR associationberlingermany[All Fields] OR associationbetween[All Fields] OR associationcantanhede[All Fields] OR associationchieri[All Fields] OR associationcolumbiamaryland[All Fields] OR associationcolumbiamarylandusa[All Fields] OR associationcta[All Fields] OR associationdagger[All Fields] OR associationdb[All Fields] OR associatione[All Fields] OR associationeducationand[All Fields] OR associationer[All Fields] OR associationes[All Fields] OR associationeuropean[All Fields] OR associationfactorsfor[All Fields] OR associationfor[All Fields] OR associationfukuoka[All Fields] OR associationg[All Fields] OR associationgranada[All Fields] OR associationhouston[All Fields] OR associationhyogojapan[All Fields] OR associationi[All Fields] OR associationin[All Fields] OR associationis[All Fields] OR associationism[All Fields] OR associationist[All Fields] OR associationistic[All Fields] OR associationists[All Fields] OR associationkibray[All Fields] OR associationlansing[All Fields] OR associationmap[All Fields] OR associationnasushiobara[All Fields] OR associationndi[All Fields] OR associationnfl[All Fields] OR associationnice[All Fields] OR associationo[All Fields] OR associationof[All Fields] OR associationopll[All Fields] OR associationpetitsprinces[All Fields] OR associationpublication[All Fields] OR associationrecams[All Fields] OR ("association"[MeSH Terms] OR "association"[All Fields] OR "associations"[All Fields]) OR associations'[All Fields] OR associations's[All Fields] OR associationsbismuth[All Fields] OR associationship[All Fields] OR associationsin[All Fields] OR associationsleffects[All Fields] OR associationsls[All Fields] OR associationsmuster[All Fields] OR associationsplay[All Fields] OR associationsstudier[All Fields] OR associationsto[All Fields] OR associationswere[All Fields] OR associationtype[All Fields] OR associationutrechtthe[All Fields] OR associationviewer[All Fields] OR associationwith[All Fields] OR associationwoods[All Fields] OR associationzurichswitzerland[All Fields] OR associatioon[All Fields] OR associatioons[All Fields] OR associatios[All Fields] OR associatiotn[All Fields] OR associatition[All Fields] OR associativa[All Fields] OR associativas[All Fields] OR associative[All Fields] OR associative'[All Fields] OR associatively[All Fields] OR associatives[All Fields] OR associativi[All Fields] OR associativiale[All Fields] OR associativism[All Fields] OR associativismo[All Fields] OR associativities[All Fields] OR associativity[All Fields] OR associativity'[All Fields] OR associativly[All Fields] OR associativo[All Fields] OR associatkd[All Fields] OR associato[All Fields] OR associaton[All Fields] OR associatonal[All Fields] OR associatons[All Fields] OR associator[All Fields] OR associator'[All Fields] OR associators[All Fields] OR associators'[All Fields] OR associatred[All Fields] OR associatrion[All Fields] OR associatron[All Fields] OR associats[All Fields] OR associatted[All Fields] OR associattion[All Fields]) OR (("family"[MeSH Terms] OR "family"[All Fields] OR "relation"[All Fields]) OR relation'[All Fields] OR relation's[All Fields] OR relationahip[All Fields] OR relational[All Fields] OR relational'[All Fields] OR relationala[All Fields] OR relationalabcom[All Fields] OR relationale[All Fields] OR relationalen[All Fields] OR relationaler[All Fields] OR relationales[All Fields] OR relationalism[All Fields] OR relationalists[All Fields] OR relationalities[All Fields] OR relationality[All Fields] OR relationally[All Fields] OR relationary[All Fields] OR relationaship[All Fields] OR relationaships[All Fields] OR relationated[All Fields] OR relationavec[All Fields] OR relationbetween[All Fields] OR relationbship[All Fields] OR relationchip[All Fields] OR relatione[All Fields] OR relationed[All Fields] OR relationeel[All Fields] OR relationele[All Fields] OR relationelle[All Fields] OR relationem[All Fields] OR relationen[All Fields] OR relationer[All Fields] OR relationerna[All Fields] OR relationerne[All Fields] OR relationes[All Fields] OR relationg[All Fields] OR relationhip[All Fields] OR relationhips[All Fields] OR relationhsip[All Fields] OR relationin[All Fields] OR relationing[All Fields] OR relationism[All Fields] OR relationismus[All Fields] OR relationist[All Fields] OR relationist'[All Fields] OR relationists[All Fields] OR relationmaking[All Fields] OR relationn[All Fields] OR relationnal[All Fields] OR relationnel[All Fields] OR relationnelle[All Fields] OR relationnelles[All Fields] OR relationnels[All Fields] OR relationnisme[All Fields] OR relationnship[All Fields] OR relationof[All Fields] OR relations[All Fields] OR relations'[All Fields] OR relations1[All Fields] OR relationsbestimmung[All Fields] OR relationschip[All Fields] OR relationserfassung[All Fields] OR relationsforstyrrelse[All Fields] OR relationsh[All Fields] OR relationshhip[All Fields] OR relationshi[All Fields] OR relationshiip[All Fields] OR relationshin[All Fields] OR relationshio[All Fields] OR relationshion[All Fields] OR relationshionship[All Fields] OR relationshiop[All Fields] OR relationshiops[All Fields] OR relationship[All Fields] OR relationship'[All Fields] OR relationship''[All Fields] OR relationship','the[All Fields] OR relationship's[All Fields] OR relationship01[All Fields] OR relationshipa[All Fields] OR relationshipa1[All Fields] OR relationshipantioxidative[All Fields] OR relationshipbetween[All Fields] OR relationshipcentred[All Fields] OR relationshipd[All Fields] OR relationshiphs[All Fields] OR relationshipjulich[All Fields] OR relationshipl[All Fields] OR relationshipls[All Fields] OR relationshipp[All Fields] OR relationshippuparia[All Fields] OR relationshipre[All Fields] OR relationships[All Fields] OR relationships'[All Fields] OR relationships1[All Fields] OR relationshipsamong[All Fields] OR relationshipsand[All Fields] OR relationshipsbetween[All Fields] OR relationshipsby[All Fields] OR relationshipslondon[All Fields] OR relationshipsmagnetic[All Fields] OR relationshipsthe[All Fields] OR relationshipswere[All Fields] OR relationshipt[All Fields] OR relationshipthe[All Fields] OR relationshipthis[All Fields] OR relationshipto[All Fields] OR relationshipwith[All Fields] OR relationshis[All Fields] OR relationshisp[All Fields] OR relationshkps[All Fields] OR relationshop[All Fields] OR relationshp[All Fields] OR relationshps[All Fields] OR relationsiip[All Fields] OR relationsinriktad[All Fields] OR relationsip[All Fields] OR relationsips[All Fields] OR relationsjhip[All Fields] OR relationsof[All Fields] OR relationspathologie[All Fields] OR relationspathologische[All Fields] OR relationspathologischen[All Fields] OR relationspathologischer[All Fields] OR relationspathologisches[All Fields] OR relationsphps[All Fields] OR relationssenkungsgeschwindigkeit[All Fields] OR relationsship[All Fields] OR relationsships[All Fields] OR relationsstructure[All Fields] OR relationssystem[All Fields] OR relationst[All Fields] OR relationstest[All Fields] OR relationstests[All Fields] OR relationsthe[All Fields] OR relationsthips[All Fields] OR relationstraning[All Fields] OR relationswhip[All Fields] OR relationthip[All Fields] OR relationto[All Fields] OR relationtships[All Fields] OR relationwith[All Fields] OR relationz[All Fields])) AND ("0001/01/01"[PDAT] : "2016/03/31"[PDAT]))** | [**5761574**](https://www.ncbi.nlm.nih.gov/pubmed/?cmd=HistorySearch&querykey=3) |
| **Final** | **#1 AND #2 AND #3** | [1883](https://www.ncbi.nlm.nih.gov/pubmed/?cmd=HistorySearch&querykey=1) |

**Supplementary Table 2. Standard rules for converting physical activity measures to standardized metric of MET-hours/week:**

| 1. When a study expressed physical activity as a specific activity (e.g., walking, cycling, gardening, etc.) and its duration, we defined the intensity of the activity according to the Compendium of Physical Activities Google Site defined by Ainsworth, et al. gardening, 5.5 METs; cycling, 7.5 METs; lifting, 6 METs; swimming, 6 METs; aerobics, 5.5 METs; jogging, 7.3 METs; golf, 4.8 METs; basketball, 6.5 METs; tennis, 5.5 METs; and brisk walking, 4.3 METs. 2. When a paper described physical activity in terms of intensity (rather than describing the actual activity that was performed) then we assume the following MET values: light intensity: 3 METs; moderate intensity: 4 METs; moderate to vigorous: 4.5; vigorous intensity: 8 METs. 3. When a paper used a range to describe an exposure (e.g. 0-2 h/week walking) we used a point estimate which was the mid-point of the range. 4. When a paper used an open category to describe an exposure we assume that the size of this category is the same as the closest equivalent exposure category and then calculate the median. For example, if a paper categorised walking as 0-2h/week; 2-4h/week; >4h/week, then we assume that the last exposure group is 4-6h/week and use the median value of 5h/week. 5. If a paper used only one open category versus the reference (e.g. > or < 3h/week versus none), we assume the size of that category is 3 + or - (half of 3) 1.5=4.5 or 1.5 h/week. 6. When a study reported only frequency of ‘sessions’ or ‘times’ or ‘occasions’ per week, we converted the frequency of physical activity per week to times/week by assigning a dose of 45 minutes per session. 7. When a study did not report the frequency of sessions per week, we assumed the following: ‘weekly’: 3 times/week; ‘several’ or ‘regular’ or ‘routinely’: 5 times/week; ‘occasionally’ or ‘sometimes’: 2 times/week. 8. Some ambiguous activities such as “exercise”, “sports” and “leisure-time physical activity” were categorised as moderate to vigorous. 9. Where a paper tried to measure amount of time spent in ‘moderate or vigorous’ physical activity but gave no information about the type of activity, we assume a MET value of 4.5. 10. Often papers categorised groups using an ‘or’ function. For example, an exposure group may consist of individuals who walk for at least 5 hours per week or exercise for at least two hours per week. In these instances, we assumed that any combination of these two activities is equally likely. So we calculated the METh/week for someone who only walks 5 hours per week, for someone who only exercises for two hours a week, and for someone who both walks for five hours a week AND exercises for two hours a week. We then calculated the average of the three measures, and assigned that value to the exposure group. 11. When a paper split groups into x-tiles of an exposure variable and provided estimates of mean and standard deviation of the exposure variable in the whole sample, we assumed that the exposure variable was log-normally distributed (unless otherwise stated in the paper) and then calculate the exposure variable for each of the x-tile variables from this assumed distribution. 12. When a physical activity level (e.g. total physical activity level) was categorized into x-tiles or groups (e.g. athletic, active, sedentary), we assume the following: highest group: vigorous intensity, 2nd highest: moderate to vigorous, 3rd highest group: moderate…. . To be converted to MET-h/week, we assumed 5 times/week for each group, and 45 minutes for each session. |
| --- |

**Supplementary Table 3. Modified Downs and Black quality checklist to assess measurement of physical activity in low back pain populations**

| Reporting |  |
| --- | --- |
| 1. Is the hypothesis/aim/objective of the study clearly described? | C/CS |
| 1. Are the main physical activity outcomes to be measured clearly described in the ‘‘Introduction’’ or ‘‘Methods’’ section? | C/CS |
| 1. Are the characteristics of the patients included in the study clearly described? | C/CS |
| 1. Are the physical activity measurements of interest clearly described? | C/CS |
| 1. Are the distributions of principal confounders in each group of subjects to be compared clearly described? | C/CS |
| 1. Are the main physical activity findings of the study clearly described? | C/CS |
| 1. Does the study provide estimates of the random variability in the physical activity data for the main LBP outcomes? | C/CS |
| 1. Have the characteristics of patients lost to follow-up been described? | C |
| 1. Have actual probability values been reported (e.g. 0.035 rather than\0.05) for the main outcomes except where the probability value is less than 0.001? | C/CS |
| External validity |  |
| 1. Were the subjects asked to participate in the study representative of the entire population from which they were recruited? | C/CS |
| 1. Were those subjects who were prepared to participate representative of the entire population from which they were recruited? | C/CS |
| Internal validity |  |
| 1. If any of the results of the study were based on ‘‘data dredging’’, was this made clear? | C/CS |
| 1. In trials and cohort studies, do the analyses adjust for different lengths of follow-up of patients, or in case–control studies, is the time period between the physical activity measurement and outcome the same for cases and controls? | C |
| 1. Were the statistical tests used to assess the relationship between the LBP outcome measure and physical activity outcomes appropriate? | C/CS |
| 1. Were the main physical activity outcome measures used accurate (valid and reliable)? | C/CS |
| Confounding (selection bias) |  |
| 1. Were the patients in different physical activity intervention groups (trials and cohort studies) or were the cases and controls (case–control studies) recruited from the same population? | C |
| 1. Were study subjects in different physical activity intervention groups (trials and cohort studies) or were the cases and controls (case–control studies) recruited over the same period of time? | C |
| 1. Was there adequate adjustment for confounding factors between physical activity and LBP in the analyses from which the main findings were drawn? | C/CS |
| 1. Were losses of patients to follow-up taken into account? | C |
| **Abbreviations:** C, cohort; CS, cross-sectional; LBP, low back pain. | |

**Supplementary Table 4. Characteristics of included studies**

| **First author (year)** | **Design** | **Study population** | **Total sample  size included in analysis** | **Age range** | **Gender (%Female)** | **LBP at baseline** | **LBP at follow-up** | **Follow-up time** | **Cases** | **Types of extracted PA** | **PA measure** |
| --- | --- | --- | --- | --- | --- | --- | --- | --- | --- | --- | --- |
| **Abolfotouh, et al. [1]** | Cross-sectional | Nurses | 254 | 25-60 | 74.8 | Prevalence of LBP in the last 12 months | N/A | N/A | 138 | Walking, running and other sports | Self-administered questionnaire |
| **Andersen, et al. [2]** | Cohort | Occupational population | 1513 | 18-67 | NR | No LBP at baseline | Prevalence of LBP in the last 12 months | 2 years | 160 | LTPA | Self-administered questionnaire |
| **Bjorck-van Dijken, et al. [3]** | Cross-sectional | General population | 5798 | 25-79 | 50.8 | Prevalence of LBP in the last week, last 6 months, and more than 6 months | N/A | N/A | 2377 | LTPA | MONICA survey |
| **Croft, et al. [4]** | Cohort | Patients registered with two general practices | 1649 | 18-75 | NR | No LBP in the previous month | Prevalence of new episodes of LBP in the past 12 months after baseline survey | 1 year | 594 | Walking, gardening and sports activities | Self-administered questionnaire |
| **Eriksen, et al. [5]** | Cohort | General population | 535 | 20-72 | 38 | No LBP in the last 12 months | Prevalence of LBP in the past 12 months | 4 years | 195 | Leisure-time physical exercise | Self-administered questionnaire |
| **Failde, et al. [6]** | Cross-sectional | University hospital employees | 890 | NR | 65.1 | Prevalence of LBP | N/A | N/A | 795 | Physical exercises | Self-administered questionnaire |
| **George, et al. [7]** | Cohort | Soldiers | 1230 | 18-35 | 30 | No previous episode of LBP lasting longer than 48 hours | Prevalence of LBP in the last month | 2 years | 518 | Exercise | Self-administered questionnaire |
| **Hartvigsen and Christensen [8]** | Cohort | Aging Danish twins | 1387 | 70-100 | 52 | No LBP during the month before the baseline survey | Prevalence of LBP in the past 12 months, any LBP in the past 12 months and LBP>30 days in the past 12 months | 2 years | 197 | Total PA | Self-administered questionnaire |
| **Heneweer, et al. [9]** | Cross-sectional | General population | 3664 | ≥25 | 55.2 | Prevalence of chronic LBP (> 3 months) in the past 12 months | N/A | N/A | 1558 | Total PA, LTPA and sport participation | SQUASH |
| **Heuch, et al. [10]** | Cohort | General population | 18,068 | 30-69 | 53 | No chronic LBP (≥3 months) in the past 12 months | Prevalence of chronic LBP (≥3 months) in the past 12 months | 11 years | 3104 | LTPA | Self-administered questionnaire |
| **Hubscher, et al. [11]** | Cross-sectional | Twins living in Australia | 486 | 18-65 | 57 | Prevalence of LBP in the past 4 weeks | N/A | N/A | 267 | Domestic, recreational PA, and recreational walking | Active Australia Survey |
| **Jacob [12]** | Cohort | Community- based random sample | 212 | 22-70 | 54.5 | No LBP in the past month | Prevalence of LBP in the previous 12 months | 1 year | 39 | LTPA and sports activities | BPAQ |
| **Junqueira, et al. [13]** | Cross-sectional | Twins living in Australia | 486 | 18-65 | 56 | Prevalence of chronic LBP in the past 4 weeks | N/A | N/A | 150 | Domestic, light walking and recreational PA | Active Australia Survey |
| **Kamada, et al. [14]** | Cross-sectional | General population | 4559 | 40-79 | 53.7 | Prevalence of chronic LBP (> 3 months) in the past 12 months | N/A | N/A | 605 | Total PA | IPAQ-SF |
| **Khruakhorn, et al. [15]** | Cross-sectional | University staff | 783 | NR | 70.7 | Prevalence of LBP in the past 6 months | N/A | N/A | 177 | Habitual PA | Self-administered questionnaire |
| **Kim, et al. [16]** | Cross-sectional | General population | 3994 | ≥50 | 55 | Prevalence of LBP (>1 month) in the past 3 months | N/A | N/A | 1100 | Moderate and vigorous activities, strength and flexibility exercises, and walking | IPAQ-SF |
| **Kwon, et al. [17]** | Cross-sectional | Patients undergoing medical examination in the center | 772 | ≥20 | 0 | Prevalence of LBP | N/A | N/A | 80 | Exercise | Precision medical questionnaire |
| **Leino-Arjas, et al. [18]** | Cohort | Metal industry employees | 546 | 18-64 | 35 | With or without LBP | Prevalence of often or frequent local LBP in the past 12 months | 28 years | 544 | LTPA | Self-administered questionnaire |
| **Levangie [19]** | Cross-sectional | Patients seeking physical therapy services | 288 | 21-50 | 60.4 | Prevalence of LBP in the past 12 months | N/A | N/A | 150 | Activity level compared with peers | Self-administered questionnaire |
| **Mandel and Lohman [20]** | Cross-sectional | Nurses | 421 | NR | NR | Prevalence of LBP > 48 hours in the past 12 months | N/A | N/A | 419 | Aerobic exercises | Self-administered questionnaire |
| **Miranda, et al. [21]** | Cohort | Workers of forest industry | 2256 | 19-67 | 26 | Without LBP in the previous 12 months | Prevalence of LBP longer than 7 days in the previous 12 months | 1 year | 474 | Physical exercise | Self-administered questionnaire |
| **Nilsen, et al. [22]** | Cohort | General population | 32417 | ≥20 | 52.7 | No chronic LBP (≥3 months) in the previous year | Prevalence of chronic LBP (≥3 months) in the past 12 months | 11 years | 3268 | Leisure-time physical exercise | Self-administered questionnaire |
| **Nordin, et al. [23]** | Cross-sectional | Health science undergraduate students | 144 | NR | 70.1 | Prevalence of LBP | N/A | N/A | 58 | Total PA | Self-administered questionnaire |
| **Nourbakhsh, et al. [24]** | Cross-sectional | Subjects selected randomly from hospitals | 840 | 20-65 | 50 | Prevalence of LBP | N/A | N/A | 420 | Level of daily exercises | Self-administered questionnaire |
| **Picavet and Schuit [25]** | Cohort | Population based study | 3759 | 20-59 | 55 | With or without LBP | Prevalence of LBP in the past 12 months | 1 to 4 years | 2018 | LTPA | Self-administered questionnaire |
| **Power, et al. [26]** | Cohort | 1958 British birth cohort | 5781 | 23 | 51 | Excluded from the analyses were those with persistent LBP at 23 and 33 years, those with incident LBP between 23 and 32 years, those with LBP at 23 years who recovered | Prevalence of LBP lasting more than one day in the past 12 months (at 32 to 33 years of age) | 10 years | 571 | Total PA (active or inactive) | Self-administered questionnaire |
| **Sandler, et al. [27]** | Cohort | Participants from previous  observational study | 4610 | 20-81 | 17 | No LBP at baseline | Prevalence of LBP | Mean of 5 years | 590 | Flexibility and muscle strengthening activities | Self-administered questionnaire |
| **Shiri, et al. [28]** | Cohort | Young Finns | 1224 | 24-39 | 52.5 | No LBP longer than 7 days in the previous 12 months | Prevalence of LBP longer than 7 days in the previous 12 months | 6 years | NR | LTPA, commuting to work by walking or cycling, participation in organised activity | Self-administered questionnaire |
| **Sitthipornvorakul, et al. [29]** | Cohort | Office workers | 387 | 20-45 | 76.2 | No LBP with pain intensity greater than 30/100 mm on Visual Analog Scale in the previous 3 months | Prevalence of LBP lasting >24 hours with pain intensity greater than 30/100 in the past month | 1 year | 244 | Walk | Digi-walker Pedometer CW700s |
| **Taanila, et al. [30]** | Cohort | Finnish male conscripts | 982 | 18-28 | 0 | Without LBP lasting longer than 1 day during the past month | Prevalence of LBP in the past 6 months | 6 months | 155 | Brisk leisure-time sport, and participation in individual aerobic sports and competitive sports | Self-administered questionnaire |
| **Thiese, et al. [31]** | Cohort | Occupational population | 68 | NR | 26.5 | No chronic LBP (>3 months) | Prevalence of LBP | 1 year | 30 | Total PA | Actigraph accelerometers model GT1M |
| **Thomas, et al. [32]** | Cohort | Patients registered with two general practices | 180 | 18-75 | 58.9 | With LBP | Prevalence of chronic (disabling) LBP | 1 year | 61 | Activity level compared with peers | Self-administered questionnaire |
| **van Oostrom, et al. [33]** | Cohort | General population | 5706 | 26-65 | 53 | Without persistent LBP (>3 months) in the previous 12 months | Prevalence of new episodes of long-standing LBP (> 3 months) during the past 12 months | 10 years | 3826-3834 | Total PA | Self-administered questionnaire |
| **Yip [34]** | Cohort | Nurses | 144 |  | 85 | Without LBP in the previous 12 months | Prevalence of LBP in the past 12 months | 1 year | 56 | LTPA | Self-administered questionnaire |
| **Zanuto, et al. [35]** | Cross-sectional | General population | 743 | ≥18 | 60.9 | Prevalence of LBP in the past 12 months | N/A | N/A | 84 | LTPA | Self-administered questionnaire |
| **Abbreviations:** BPAQ, Baecke Physical Activity Questionnaire; IPAQ-SF, International Physical Activity Questionnaire-Short Form; LBP, Low Back Pain; LTPA, Leisure-Time Physical Activity; N/A, Not Applicable; NR, Not Reported; PA, Physical Activity; SQUASH, Short Questionnaire to Assess Health enhancing physical activity. | | | | | | | | | | | |

**Supplementary Table 5. Studies included in the meta-analyses**

| **First author (year)** | **Design** | **Study population** | **Total sample  size included in analysis** | **Age range** | **Gender (%Female)** | **LBP at baseline** | **LBP at follow-up** | **Follow-up time** | **Cases** | **Types of extracted PA** | **PA measure** | **Adjusted confounding factors** |
| --- | --- | --- | --- | --- | --- | --- | --- | --- | --- | --- | --- | --- |
| **Abolfotouh, et al. [1]** | Cross-sectional | Nurses | 254 | 25-60 | 74.8 | Prevalence of LBP in the last 12 months | N/A | N/A | 138 | Walking, running and other sports | Self-administered questionnaire | Age, arthritis, obesity and diabetes |
| **Andersen, et al. [2]** | Cohort | Occupational population | 1513 | 18-67 | NR | No LBP at baseline | Prevalence of LBP in the last 12 months | 2 years | 160 | LTPA | Self-administered questionnaire | Age, gender, occupational group, and intervention group |
| **Bjorck-van Dijken, et al. [3]** | Cross-sectional | General population | 5798 | 25-79 | 50.8 | Prevalence of LBP in the last week, last 6 months, and more than 6 months | N/A | N/A | 2377 | LTPA | MONICA survey | Age, gender BMI, education, regular smoking, and community size |
| **Croft, et al. [4]** | Cohort | Patients registered with two general practices | 1649 | 18-75 | NR | No LBP in the previous month | Prevalence of new episodes of LBP in the past 12 months after baseline survey | 1 year | 594 | Walking, gardening and sports activities | Self-administered questionnaire | Age and gender |
| **Eriksen, et al. [5]** | Cohort | General population | 535 | 20-72 | 38 | No LBP in the last 12 months | Prevalence of LBP in the past 12 months | 4 years | 195 | Leisure-time physical exercise | Self-administered questionnaire | Age, gender, marital status,  smoking, heavy physical work, emotional symptoms,  musculoskeletal pain other than low back pain, and monotonous movements in  the job |
| **George, et al. [7]** | Cohort | Soldiers | 1230 | 18-35 | 30 | No previous episode of LBP lasting longer than 48 hours | Prevalence of LBP in the last month | 2 years | 518 | Exercise | Self-administered questionnaire | Age, gender, BMI, race, education, income, active duty status, smoking, time in army, previous injury, depression, anxiety, fear of pain questionnaire, exercise and education groups |
| **Heneweer, et al. [9]** | Cross-sectional | General population | 3664 | ≥25 | 55.2 | Prevalence of chronic LBP (> 3 months) in the past 12 months | N/A | N/A | 1558 | Total PA, LTPA and sport participation | SQUASH | Age and gender |
| **Heuch, et al. [10]** | Cohort | General population | 18,068 | 30-69 | 53 | No chronic LBP (≥3 months) in the past 12 months | Prevalence of chronic LBP (≥3 months) in the past 12 months | 11 years | 3104 | LTPA | Self-administered questionnaire | Gender, BMI, education, employment, occupational activity and smoking |
| **Hubscher, et al. [11]** | Cross-sectional | Twins living in Australia | 486 | 18-65 | 57 | Prevalence of LBP in the past 4 weeks | N/A | N/A | 267 | Domestic, recreational PA, and recreational walking | Active Australia Survey | Age, gender and smoking |
| **Kamada, et al. [14]** | Cross-sectional | General population | 4559 | 40-79 | 53.7 | Prevalence of chronic LBP (> 3 months) in the past 12 months | N/A | N/A | 605 | Total PA | IPAQ-SF | Age, gender, BMI, education years, self-rated health, chronic disease history, depressive symptom, smoking, history of joint injuries and pain management |
| **Khruakhorn, et al. [15]** | Cross-sectional | University staff | 783 | NR | 70.7 | Prevalence of LBP in the past 6 months | N/A | N/A | 177 | Habitual PA | Self-administered questionnaire | Gender, nutritional level, and activity at work |
| **Kim, et al. [16]** | Cross-sectional | General population | 3994 | ≥50 | 55 | Prevalence of LBP (>1 month) in the past 3 months | N/A | N/A | 1100 | Moderate and vigorous activities, strength and flexibility exercises, and walking | IPAQ-SF | Age, gender, BMI, total hip osteoporosis status, education, residential area, smoking, and alcohol consumption |
| **Kwon, et al. [17]** | Cross-sectional | Patients undergoing medical examination in the center | 772 | ≥20 | 0 | Prevalence of LBP | N/A | N/A | 80 | Exercise | Precision medical questionnaire | Age, BMI, educational level, smoking and level of stress |
| **Leino-Arjas, et al. [18]** | Cohort | Metal industry employees | 546 | 18-64 | 35 | With or without LBP | Prevalence of often or frequent local LBP in the past 12 months | 28 years | 544 | LTPA | Self-administered questionnaire | Age, gender, and occupational class |
| **Miranda, et al. [21]** | Cohort | Workers of forest industry | 2256 | 19-67 | 26 | Without LBP in the previous 12 months | Prevalence of LBP longer than 7 days in the previous 12 months | 1 year | 474 | Physical exercise | Self-administered questionnaire | Age, gender, BMI, smoking, heavy lifting, awkward postures, and whole body vibration |
| **Nilsen, et al. [22]** | Cohort | General population | 32417 | ≥20 | 52.7 | No chronic LBP (≥3 months) in the previous year | Prevalence of chronic LBP (≥3 months) in the past 12 months | 11 years | 3268 | Leisure-time physical exercise | Self-administered questionnaire | Age, gender, BMI, smoking and occupation |
| **Picavet and Schuit [25]** | Cohort | Population based study | 3759 | 20-59 | 55 | With or without LBP | Prevalence of LBP in the past 12 months | 1 to 4 years | 2018 | LTPA | Self-administered questionnaire | Age, gender, and LBP at  baseline |
| **Sandler, et al. [27]** | Cohort | Participants from previous  observational study | 4610 | 20-81 | 17 | No LBP at baseline | Prevalence of LBP | Mean of 5 years | 590 | Flexibility and muscle strengthening activities | Self-administered questionnaire | Age and gender |
| **Shiri, et al. [28]** | Cohort | Young Finns | 1224 | 24-39 | 52.5 | No LBP longer than 7 days in the previous 12 months | Prevalence of LBP longer than 7 days in the previous 12 months | 6 years | NR | LTPA, commuting to work by walking or cycling, participation in organised activity | Self-administered questionnaire | BMI, total aerobics activity , current smoking, hypertension, diabetes, hypercholesterolemia, ,cardiorespiratory fitness and flexibility activity |
| **Taanila, et al. [30]** | Cohort | Finnish male conscripts | 982 | 18-28 | 0 | Without LBP lasting longer than 1 day during the past month | Prevalence of LBP in the past 6 months | 6 months | 155 | Brisk leisure-time sport, and participation in individual aerobic sports and competitive sports | Self-administered questionnaire | Age, company, smoking, baseline medical conditions (sports injury, sum factor of earlier musculoskeletal symptoms, regular medication,  chronic impairment or disability because of prior musculoskeletal injury, and orthopedic surgery), educational level, school degree level, father’s occupation,  and participating in individual aerobic sports (12 adjusting variables) |
| **Thiese, et al. [31]** | Cohort | Occupational population | 68 | NR | 26.5 | No chronic LBP (>3 months) | Prevalence of LBP | 1 year | 30 | Total PA | Actigraph accelerometers model GT1M | Age, gender, BMI, smoking, compressive force, depression, and seeing a health care provider for LBP |
| **Thomas, et al. [32]** | Cohort | Patients registered with two general practices | 180 | 18-75 | 58.9 | With LBP | Prevalence of chronic (disabling) LBP | 1 year | 61 | Activity level compared with peers | Self-administered questionnaire | Age and gender |
| **van Oostrom, et al. [33]** | Cohort | General population | 5706 | 26-65 | 53 | Without persistent LBP (>3 months) in the previous 12 months | Prevalence of new episodes of long-standing LBP (> 3 months) during the past 12 months | 10 years | 3826-3834 | Total PA | Self-administered questionnaire | Age, gender, BMI, smoking, educational level, and work status |
| **Zanuto, et al. [35]** | Cross-sectional | General population | 743 | ≥18 | 60.9 | Prevalence of LBP in the past 12 months | N/A | N/A | 84 | LTPA | Self-administered questionnaire | Socioeconomic variables, behavioral variables and overweight |
| **Abbreviations:** BMI, Body Mass Index; IPAQ-SF, International Physical Activity Questionnaire-Short Form; LBP, Low Back Pain; LTPA, Leisure-Time Physical Activity; N/A, Not Applicable, NR, Not Reported; PA, Physical Activity; SQUASH: Short Questionnaire to Assess Health enhancing physical activity. | | | | | | | | | | | | |

**Supplementary Table 6. Methodological quality assessment of included studies**

| 1. **Methodological quality assessment of cohort studies** | | | | | | | | | | | | | | | | | | | | | |
| --- | --- | --- | --- | --- | --- | --- | --- | --- | --- | --- | --- | --- | --- | --- | --- | --- | --- | --- | --- | --- | --- |
| **First author (year)** | **1** | **2** | **3** | **4** | **5** | **6** | **7** | **8** | **9** | **10** | **11** | **12** | **13** | **14** | **15** | **16** | **17** | **18** | **19** | **Total score (19)** |  |
| **Andersen, et al. [2]** | 1 | 0 | 1 | 0 | 1 | 1 | 1 | 1 | 0 | 1 | 1 | 1 | 1 | 1 | 0 | 1 | 1 | 1 | 1 | **15** |  |
| **Croft, et al. [4]** | 1 | 1 | 1 | 0 | 1 | 1 | 1 | 1 | 0 | 1 | 1 | 1 | 1 | 1 | 0 | 1 | 1 | 1 | 1 | **16** |  |
| **Eriksen, et al. [5]** | 1 | 1 | 1 | 1 | 1 | 1 | 1 | 1 | 0 | 1 | 1 | 1 | 1 | 1 | 0 | 1 | 1 | 1 | 1 | **17** |  |
| **George, et al. [7]** | 1 | 0 | 1 | 0 | 1 | 0 | 1 | 1 | 1 | 0 | 0 | 1 | 1 | 1 | 0 | 1 | 1 | 1 | 1 | **13** |  |
| **Hartvigsen and Christensen [8]** | 1 | 1 | 1 | 1 | 1 | 1 | 1 | 0 | 0 | 1 | 0 | 1 | 1 | 1 | 1 | 1 | 1 | 1 | 1 | **16** |  |
| **Heuch, et al. [10]** | 1 | 1 | 0 | 1 | 1 | 1 | 1 | 0 | 0 | 1 | 0 | 1 | 1 | 1 | 1 | 1 | 1 | 1 | 1 | **15** |  |
| **Jacob [12]** | 1 | 1 | 1 | 1 | 0 | 0 | 1 | 1 | 0 | 1 | 1 | 1 | 1 | 1 | 1 | 1 | 1 | 0 | 1 | **15** |  |
| **Leino-Arjas, et al. [18]** | 1 | 1 | 1 | 1 | 1 | 1 | 1 | 0 | 0 | 0 | 0 | 1 | 1 | 1 | 1 | 1 | 1 | 1 | 0 | **14** |  |
| **Miranda, et al. [21]** | 1 | 1 | 1 | 0 | 1 | 1 | 1 | 0 | 0 | 1 | 1 | 1 | 1 | 1 | 0 | 1 | 1 | 1 | 0 | **14** |  |
| **Nilsen, et al. [22]** | 1 | 1 | 1 | 1 | 1 | 1 | 1 | 0 | 1 | 1 | 1 | 1 | 1 | 1 | 0 | 1 | 1 | 1 | 0 | **16** |  |
| **Picavet and Schuit [25]** | 1 | 1 | 0 | 1 | 0 | 1 | 0 | 0 | 0 | 1 | 0 | 1 | 0 | 1 | 1 | 1 | 1 | 1 | 0 | **11** |  |
| **Power, et al. [26]** | 1 | 0 | 1 | 0 | 0 | 0 | 1 | 0 | 0 | 1 | 0 | 1 | 1 | 1 | 0 | 1 | 1 | 0 | 0 | **9** |  |
| **Sandler, et al. [27]** | 1 | 1 | 1 | 1 | 1 | 1 | 1 | 0 | 1 | 0 | 0 | 1 | 1 | 1 | 0 | 1 | 1 | 1 | 0 | **14** |  |
| **Shiri, et al. [28]** | 1 | 1 | 1 | 1 | 1 | 1 | 1 | 0 | 0 | 0 | 0 | 1 | 1 | 1 | 0 | 1 | 1 | 1 | 0 | **13** |  |
| **Sitthipornvorakul, et al. [29]** | 1 | 1 | 1 | 1 | 0 | 1 | 1 | 0 | 0 | 0 | 0 | 1 | 1 | 1 | 1 | 1 | 1 | 1 | 0 | **13** |  |
| **Taanila, et al. [30]** | 1 | 1 | 1 | 1 | 1 | 1 | 1 | 0 | 0 | 1 | 1 | 1 | 1 | 1 | 0 | 1 | 1 | 1 | 1 | **16** |  |
| **Thiese, et al. [31]** | 1 | 1 | 1 | 1 | 1 | 1 | 1 | 1 | 0 | 0 | 0 | 1 | 1 | 1 | 1 | 1 | 1 | 1 | 1 | **16** |  |
| **Thomas, et al. [32]** | 1 | 1 | 1 | 0 | 1 | 0 | 1 | 1 | 0 | 1 | 1 | 1 | 1 | 1 | 0 | 1 | 1 | 0 | 1 | **14** |  |
| **van Oostrom, et al. [33]** | 1 | 1 | 1 | 0 | 1 | 0 | 1 | 0 | 0 | 1 | 1 | 1 | 1 | 1 | 0 | 1 | 1 | 1 | 1 | **14** |  |
| **Yip [34]** | 1 | 1 | 1 | 1 | 0 | 1 | 1 | 1 | 1 | 0 | 0 | 1 | 1 | 1 | 0 | 1 | 1 | 0 | 0 | **13** |  |
| **Score interpretation:** 1=Yes; 0= No or Unable to determine. | | | | | | | | | | | | | | | | | | | | |  |

| 1. **Methodological quality assessment of cross-sectional studies** | | | | | | | | | | | | | | | | | | | | |
| --- | --- | --- | --- | --- | --- | --- | --- | --- | --- | --- | --- | --- | --- | --- | --- | --- | --- | --- | --- | --- |
| **First author (year)** | **1** | **2** | **3** | **4** | **5** | **6** | **7** | **8** | **9** | **10** | **11** | **12** | **13** | **14** | **15** | **16** | **17** | **18** | **19** | **Total score (14)** |
| **Abolfotouh, et al. [1]** | 1 | 1 | 1 | 0 | 0 | 1 | 1 |  | 1 | 1 | 1 | 0 |  | 1 | 0 |  |  | 0 |  | **9** |
| **Bjorck-van Dijken, et al. [3]** | 1 | 1 | 1 | 1 | 1 | 1 | 1 |  | 0 | 1 | 0 | 1 |  | 1 | 1 |  |  | 1 |  | **12** |
| **Failde, et al. [6]** | 1 | 0 | 1 | 0 | 1 | 1 | 1 |  | 0 | 1 | 1 | 0 |  | 1 | 0 |  |  | 0 |  | **8** |
| **Heneweer, et al. [9]** | 1 | 1 | 1 | 1 | 0 | 1 | 1 |  | 0 | 1 | 1 | 1 |  | 1 | 1 |  |  | 0 |  | **11** |
| **Hubscher, et al. [11]** | 1 | 1 | 1 | 1 | 0 | 1 | 1 |  | 1 | 1 | 0 | 1 |  | 1 | 1 |  |  | 1 |  | **12** |
| **Junqueira, et al. [13]** | 1 | 1 | 1 | 1 | 1 | 1 | 1 |  | 1 | 0 | 1 | 1 |  | 1 | 1 |  |  | 1 |  | **13** |
| **Kamada, et al. [14]** | 1 | 1 | 1 | 1 | 1 | 1 | 1 |  | 1 | 1 | 1 | 1 |  | 1 | 1 |  |  | 1 |  | **14** |
| **Khruakhorn, et al. [15]** | 1 | 1 | 1 | 1 | 1 | 1 | 1 |  | 1 | 0 | 0 | 1 |  | 1 | 1 |  |  | 1 |  | **12** |
| **Kim, et al. [16]** | 1 | 1 | 1 | 1 | 1 | 1 | 1 |  | 1 | 1 | 0 | 1 |  | 1 | 1 |  |  | 1 |  | **13** |
| **Kwon, et al. [17]** | 1 | 1 | 1 | 0 | 0 | 1 | 1 |  | 1 | 1 | 0 | 1 |  | 1 | 0 |  |  | 1 |  | **10** |
| **Levangie [19]** | 1 | 0 | 1 | 0 | 1 | 1 | 1 |  | 0 | 1 | 0 | 1 |  | 1 | 0 |  |  | 0 |  | **8** |
| **Mandel and Lohman [20]** | 1 | 0 | 1 | 0 | 0 | 1 | 1 |  | 1 | 1 | 0 | 1 |  | 1 | 0 |  |  | 0 |  | **8** |
| **Nordin, et al. [23]** | 1 | 0 | 1 | 0 | 0 | 1 | 0 |  | 1 | 0 | 0 | 1 |  | 1 | 0 |  |  | 0 |  | **6** |
| **Nourbakhsh, et al. [24]** | 1 | 0 | 1 | 0 | 0 | 1 | 0 |  | 1 | 0 | 0 | 0 |  | 1 | 0 |  |  | 0 |  | **5** |
| **Zanuto, et al. [35]** | 1 | 1 | 1 | 1 | 1 | 1 | 1 |  | 0 | 1 | 0 | 0 |  | 1 | 1 |  |  | 1 |  | **11** |
| **Score interpretation:** 1=Yes; 0= No or Unable to determine. **Note:** highlighted columns are not applicable for cross-sectional studies. | | | | | | | | | | | | | | | | | | | | |

**Supplementary Table 7. Comparison groups included in the meta-analyses**

| 1. **Medium level versus low level physical activity (cohort studies)** | | | | |
| --- | --- | --- | --- | --- |
|  | **Physical activity** | | | |
| **Author** | **Type** | **PA level, intensity, frequency and/or duration** | **Assigned dose in MET-hours/week** | **Reference category** |
| Croft, et al. [4] (Men) (a) | Gardening | Weekly | 12.37 | Never |
| Croft, et al. [4] (Men) (b) | Sports activities | Regular | 16.87 | Not regular |
| Croft, et al. [4] (Women) (a) | Gardening | Weekly | 12.37 | Never |
| Croft, et al. [4] (Women) (b) | Sports activities | Regular | 16.87 | Not regular |
| George, et al. [7] | Exercise routinely | Yes | 16.87 | NO |
| Heuch, et al. [10] (Men) | LTPA | Hard 1-2 hours/week | 12 | Light <1 hour/week |
| Heuch, et al. [10] (Women) | LTPA | Hard 1-2 hours/week | 12 | Light <1 hour/week |
| Leino-Arjas, et al. [18] | LTPA | Intermediate tertile | 16.87 | Lowest tertile |
| Nilsen, et al. [22] (Men) | Leisure-time physical exercises | 1-1.9 hours/week | 13.5 | No activity or <1session/week |
| Nilsen, et al. [22] (Women) | Leisure-time physical exercises | 1-1.9 hours/week | 13.5 | No activity or <1session/week |
| Taanila, et al. [30] | Participation in competitive sports | Yes | 12 | None |
| Thomas, et al. [32] | Total PA | More/same | 16.87 | Less |
| **Abbreviations:** PA, Physical Activity; LTPA, Leisure-Time Physical Activity. | | | |  |

| 1. **Medium level versus low level physical activity (cross-sectional studies)** | | | | |
| --- | --- | --- | --- | --- |
|  | **Physical activity** | | | |
| **Author** | **Type** | **PA level, intensity, frequency and/or duration** | **Assigned dose in MET-hours/week** | **Reference category** |
| Abolfotouh, et al. [1] | Running | >15min/day | 18.74 | <15 min/day |
| Hubscher, et al. [11] | Recreational PA | Moderate/vigorous (≥2hrs/week) | 13.5 | No moderate PA |
| Khruakhorn, et al. [15] | Habitual PA level | Active | 16.87 | Sedentary |
| Kim, et al. [16] (Men) | Vigorous PA | Vigorous PA (at least 20 minutes of vigorous activity on 3 or more days per week) | 18 | No |
| Kim, et al. [16] (Women) | Vigorous PA | Vigorous PA (at least 20 minutes of vigorous activity on 3 or more days per week) | 18 | No |
| Kwon, et al. [17] | Exercise frequency | ≥5-6 times/week | 18.56 | None |
| Zanuto, et al. [35] | LTPA | ≥180 min/week | 20.25 | <180 min/week |
| **Abbreviations:** PA, Physical Activity; LTPA, Leisure-Time Physical Activity. | | | |  |

| 1. **High level versus low level physical activity (cohort studies)** | | | | |
| --- | --- | --- | --- | --- |
|  | **Physical activity** | | | |
| **Author** | **Type** | **PA level, intensity, frequency and/or duration** | **Assigned dose in MET-hours/week** | **Reference category** |
| Andersen, et al. [2] | LTPA | High level (light activity > 4 hours/ week or 2–4 hours of hard physical activity or hard physical activity for > 4 hours/week) | 30 | Low level (almost none or light activity for < 2 hours/week or light activity for 2–4 hours/week) |
| Croft, et al. [4] (Men) | Walking | >30 min/day | 22.57 | <30 min/day |
| Croft, et al. [4] (Women) | Walking | >30 min/day | 22.57 | <30 min/day |
| Heuch, et al. [10] (Men) | LTPA | Hard ≥ 3 hours/week | 36 | Light <1 hour/week |
| Heuch, et al. [10] (Women) | LTPA | Hard ≥ 3 hours/week | 36 | Light <1 hour/week |
| Leino-Arjas, et al. [18] | LTPA | High level | 30 | Lowest tertile |
| Sandler, et al. [27] (a) | Muscle-strengthening activities | Yes (mean=1462 MET-minutes/wk) | 24.37 | No (mean=872 MET-minutes/week) |
| Sandler, et al. [27] (b) | Flexibility activities | Yes (mean=1471 MET-minutes/wk) | 24.52 | No (mean=936 MET-minutes/week) |
| Shiri, et al. [28] | LTPA | Highest tertile ( METs > 40/ week) | 60 | Moderate level (METs 10-40/week) |
| Taanila, et al. [30] | Brisk leisure-time sport (sweating exercise) | ≥ 3 times/week | 27 | None |
| Thiese, et al. [31] (a) | Tertiles of total minutes per day of moderate or vigorous activity | Highest tertile (mean=181.96 min/day) | 95.53 | Middle tertile (mean= 108.59) |
| Thiese, et al. [31] (b) | Tertiles of total minutes per day of light activity | Highest tertile (mean= 179.57 min/day) | 62.85 | Middle tertile (mean= 141.57) |
| van Oostrom, et al. [33] | Physical activity level | Active (≥3.5 h per week spent on cycling, gardening or sports activities with MET≥4.0) | 21 | Inactive |
| **Abbreviations:** PA, Physical Activity; LTPA, Leisure-Time Physical Activity. | | | |  |

| 1. **High level versus low level physical activity (cross-sectional studies)** | | | | |
| --- | --- | --- | --- | --- |
|  | **Physical activity** | | | |
| **Author** | **Type** | **PA level, intensity, frequency and/or duration** | **Assigned dose in MET-hours/week** | **Reference category** |
| Bjorck-van Dijken, et al. [3] | LTPA | Highest level (ranged from moderate physical activity 1–2 h a week to practicing sports at a competitive level several times a week) | 29.25 | Lowest level (ranged from no physical activity at all to light physical training with a minimum of 2 h a week) |
| Heneweer, et al. [9] | Total PA | High (representing physical activity levels within the highest quartile of the amount of physical activity and the performance of high intensive sport activities (≥ 50 MET hours)) | 75 | Moderate |
| Hubscher, et al. [11] | Domestic PA | Heavy (≥2hrs/week) | 24 | Not heavy |
| Kamada, et al. [14] (a) | Total PA | 23.1-75.3 MET-hours/week | 49.2 | 8.25-23 MET-hours/week |
| Kamada, et al. [14] (b) | Total PA | ≥75.4 MET-hours/week | 101.5 | 8.25-23 MET-hours/week |
| Khruakhorn, et al. [15] | Total PA | Athletic | 30 | Sedentary |
| Kim, et al. [16] (Men) (a) | Moderate PA | Moderate (at least 30 minutes of moderate-intensity activity on 5 or more days per week) | 21 | No |
| Kim, et al. [16] (Men) (b) | Walking | Walking at least 30 minutes of walking on 5 or more days per week. | 22.57 | No |
| Kim, et al. [16] (Women) (a) | Moderate PA | Moderate (at least 30 minutes of moderate-intensity activity on 5 or more days per week) | 21 | No |
| Kim, et al. [16] (Women) (b) | Walking | Walking at least 30 minutes of walking on 5 or more days per week. | 22.57 | No |
| **Abbreviations:** PA, Physical Activity; LTPA, Leisure-Time Physical Activity. | | | |  |

| 1. **Medium level versus low level leisure-time physical activity (cohort studies)** | | | | |
| --- | --- | --- | --- | --- |
|  | **Leisure-time physical activity** | | | |
| **Author** | **Type** | **PA level, intensity, frequency and/or duration** | **Assigned dose in MET-hours/week** | **Reference category** |
| Croft, et al. [4] (Men) | Sports activities | Regular | 16.87 | Not regular |
| Croft, et al. [4] (Women) | Sports activities | Regular | 16.87 | Not regular |
| George, et al. [7] | Exercise routinely | Yes | 16.87 | No |
| Heuch, et al. [10] (Men) | LTPA | Hard 1-2 hours/week | 12 | Light <1 hour/week |
| Heuch, et al. [10] (Women) | LTPA | Hard 1-2 hours/week | 12 | Light <1 hour/week |
| Leino-Arjas, et al. [18] | LTPA | Intermediate tertile | 16.87 | Lowest tertile |
| Nilsen, et al. [22] (Men) | Leisure-time physical exercises | 1-1.9 hours/week | 13.5 | No activity or <1 session/week |
| Nilsen, et al. [22] (Women) | Leisure-time physical exercises | 1-1.9 hours/week | 13.5 | No activity or <1 session/week |
| Taanila, et al. [30] | Participation in competitive sports | Yes | 12 | None |
| **Abbreviations:** PA, Physical Activity; LTPA, Leisure-Time Physical Activity. | | | |  |

| 1. **Medium level versus low level leisure-time physical activity (cross-sectional studies)** | | | | |
| --- | --- | --- | --- | --- |
|  | **Leisure-time physical activity** | | | |
| **Author** | **Type** | **PA level, intensity, frequency and/or duration** | **Assigned dose in MET-hours/week** | **Reference category** |
| Heneweer, et al. [9] | Sport participation | >2.5 hours/week | 14.62 | <1 hour/week |
| Hubscher, et al. [11] | Recreational PA | Moderate/vigorous (≥2hrs/week) | 13.5 | No moderate PA |
| Kim, et al. [16] (Men) (a) | Strength exercises | ≥2 days/week | 11.81 | <2 days/week |
| Kim, et al. [16] (Men) (b) | Flexibility exercises | ≥2 days/week | 11.81 | <2 days/week |
| Kim, et al. [16] (Women) (a) | Strength exercises | ≥2 days/week | 11.81 | <2 days/week |
| Kim, et al. [16] (Women) (b) | Flexibility exercises | ≥2 days/week | 11.81 | <2 days/week |
| Kwon, et al. [17] (a) | Exercise frequency | 3-4 times/week | 11.81 | None |
| Kwon, et al. [17] (b) | Exercise frequency | ≥5-6 times/week | 18.56 | None |
| **Abbreviations:** PA, Physical Activity; LTPA, Leisure-Time Physical Activity. | | | |  |

| 1. **High level versus low level leisure-time physical activity (cohort studies)** | | | | |
| --- | --- | --- | --- | --- |
|  | **Leisure-time physical activity** | | | |
| **Author** | **Type** | **PA level, intensity, frequency and/or duration** | **Assigned dose in MET-hours/week** | **Reference category** |
| Andersen, et al. [2] | LTPA | High level (light activity > 4 hours/ week or 2–4 hours of hard physical activity or hard physical activity for > 4 hours/week) | 30 | Low level (almost none or light activity for < 2 hours/week or light activity for 2–4 hours/week) |
| Croft, et al. [4] (Men) | Walking | >30 min/day | 22.57 | <30 min/day |
| Croft, et al. [4] (Women) | Walking | >30 min/day | 22.57 | <30 min/day |
| Heuch, et al. [10] (Men) | LTPA | Hard ≥ 3 hours/week | 36 | Light <1 hour/week |
| Heuch, et al. [10] (Women) | LTPA | Hard ≥ 3 hours/week | 36 | Light <1 hour/week |
| Leino-Arjas, et al. [18] | LTPA | Highest tertile | 30 | Lowest tertile |
| Sandler, et al. [27] (a) | Muscle-strengthening activities | Yes (mean=1462 MET-minutes/week) | 24.37 | No (mean=872 MET-minutes/week) |
| Sandler, et al. [27] (b) | Flexibility activities | Yes (mean=1471 MET-minutes/week) | 24.52 | No (mean=936 MET-minutes/week) |
| Shiri, et al. [28] | LTPA | Highest tertile (> 40 METs / week) | 60 | Moderate level (METs 10-40) |
| Taanila, et al. [30] | Brisk leisure-time sport (sweating exercise) | ≥ 3 times/week | 27 | No physical exercise |
| **Abbreviations:** PA, Physical Activity; LTPA, Leisure-Time Physical Activity. | | | |  |

| 1. **High level versus low level leisure-time physical activity (cross-sectional studies)** | | | | |
| --- | --- | --- | --- | --- |
|  | **Leisure-time physical activity** | | | |
| **Author** | **Type** | **PA level, intensity, frequency and/or duration** | **Assigned dose in MET-hours/week** | **Reference category** |
| Abolfotouh, et al. [1] | Running | >15min/day | 18.74 | <15 min/day |
| Bjorck-van Dijken, et al. [3] | LTPA | Highest level (ranged from moderate physical activity 1–2 h a week to practicing sports at a competitive level several times a week) | 29.25 | Lowest level (ranged from no physical activity at all to light physical training with a minimum of 2 h a week) |
| Heneweer, et al. [9] (a) | LTPA | 50% (6 hours/week) | 27 | 25% (2 hours/week) |
| Heneweer, et al. [9] (b) | LTPA | 75% (11 hours/week) | 49.5 | 25% (2 hours/week) |
| Heneweer, et al. [9] (c) | LTPA | >75% (14 hours/week) | 63 | 25% (2 hours/week) |
| Kim, et al. [16] (Men) | Walking | Walking at least 30 minutes of walking on 5 or more days per week. | 22.57 | No |
| Kim, et al. [16] (Women) | Walking | Walking at least 30 minutes of walking on 5 or more days per week. | 22.57 | No |
| Zanuto, et al. [35] | LTPA | ≥180 min/week | 20.25 | <180 min/week |
| **Abbreviations:** PA, Physical Activity; LTPA, Leisure-Time Physical Activity. | | | |  |

**References**

1. Abolfotouh SM, Mahmoud K, Faraj K, et al. (2015) Prevalence, consequences and predictors of low back pain among nurses in a tertiary care setting. Int Orthop. doi: 10.1007/s00264-015-2900-x

2. Andersen JH, Haahr JP, Frost P (2007) Risk factors for more severe regional musculoskeletal symptoms: a two-year prospective study of a general working population. Arthritis Rheum 56:1355-1364. doi: 10.1002/art.22513

3. Bjorck-van Dijken C, Fjellman-Wiklund A, Hildingsson C (2008) Low back pain, lifestyle factors and physical activity: a population based-study. J Rehabil Med 40:864-869. doi: 10.2340/16501977-0273

4. Croft PR, Papageorgiou AC, Thomas E, et al. (1999) Short-term physical risk factors for new episodes of low back pain. Prospective evidence from the South Manchester Back Pain Study. Spine (Phila Pa 1976) 24:1556-1561

5. Eriksen W, Natvig B, Bruusgaard D (1999) Smoking, heavy physical work and low back pain: a four-year prospective study. Occup Med (Lond) 49:155-160

6. Failde I, Gonzalez JL, Novalbos JP, et al. (2000) Psychological and occupational predictive factors for back pain among employees of a university hospital in southern Spain. Occup Med (Lond) 50:591-596

7. George SZ, Childs JD, Teyhen DS, et al. (2012) Predictors of Occurrence and Severity of First Time Low Back Pain Episodes: Findings from a Military Inception Cohort. PLoS One 7:e30597. doi: 10.1371/journal.pone.0030597

8. Hartvigsen J, Christensen K (2007) Active lifestyle protects against incident low back pain in seniors: a population-based 2-year prospective study of 1387 Danish twins aged 70-100 years. Spine (Phila Pa 1976) 32:76-81. doi: 10.1097/01.brs.0000250292.18121.ce

9. Heneweer H, Vanhees L, Picavet HSJ (2009) Physical activity and low back pain: A U-shaped relation? Pain 143:21-25

10. Heuch I, Heuch I, Hagen K, et al. (2016) Is there a U-shaped relationship between physical activity in leisure time and risk of chronic low back pain? A follow-up in the HUNT Study. BMC Public Health 16:306. doi: 10.1186/s12889-016-2970-8

11. Hubscher M, Ferreira ML, Junqueira DR, et al. (2014) Heavy domestic, but not recreational, physical activity is associated with low back pain: Australian Twin low BACK pain (AUTBACK) study. Eur Spine J 23:2083-2089. doi: 10.1007/s00586-014-3258-2

12. Jacob T (2006) Low back pain incident episodes: a community-based study. The Spine Journal 6:306-310. doi: 10.1016/j.spinee.2005.06.011

13. Junqueira DR, Ferreira ML, Refshauge K, et al. (2014) Heritability and lifestyle factors in chronic low back pain: results of the Australian twin low back pain study (The AUTBACK study). Eur J Pain 18:1410-1418. doi: 10.1002/ejp.506

14. Kamada M, Kitayuguchi J, Lee IM, et al. (2014) Relationship between physical activity and chronic musculoskeletal pain among community-dwelling Japanese adults. J Epidemiol 24:474-483

15. Khruakhorn S, Sritipsukho P, Siripakarn Y, et al. (2010) Prevalence and risk factors of low back pain among the university staff. J Med Assoc Thai 93 Suppl 7:S142-148

16. Kim W, Jin YS, Lee CS, et al. (2014) Relationship between the type and amount of physical activity and low back pain in Koreans aged 50 years and older. PM R 6:893-899. doi: 10.1016/j.pmrj.2014.04.009

17. Kwon MA, Shim WS, Kim MH, et al. (2006) A correlation between low back pain and associated factors: a study involving 772 patients who had undergone general physical examination. J Korean Med Sci 21:1086-1091. doi: 10.3346/jkms.2006.21.6.1086

18. Leino-Arjas P, Solovieva S, Kirjonen J, et al. (2006) Cardiovascular risk factors and low-back pain in a long-term follow-up of industrial employees. Scand J Work Environ Health 32:12-19. doi: 10.5271/sjweh.971

19. Levangie PK (1999) Association of low back pain with self-reported risk factors among patients seeking physical therapy services. Phys Ther 79:757-766

20. Mandel JH, Lohman W (1987) Low back pain in nurses: the relative importance of medical history, work factors, exercise, and demographics. Res Nurs Health 10:165-170

21. Miranda H, Viikari-Juntura E, Punnett L, et al. (2008) Occupational loading, health behavior and sleep disturbance as predictors of low-back pain. Scand J Work Environ Health 34:411-419. doi: DOI 10.5271/sjweh.1290

22. Nilsen TI, Holtermann A, Mork PJ (2011) Physical exercise, body mass index, and risk of chronic pain in the low back and neck/shoulders: longitudinal data from the Nord-Trondelag Health Study. Am J Epidemiol 174:267-273. doi: 10.1093/aje/kwr087

23. Nordin NAM, Singh DKA, Kanglun L (2014) Low Back Pain and Associated Risk Factors among Health Science Undergraduates. Sains Malaysiana 43:423-428

24. Nourbakhsh MR, Moussavi SJ, Salavati M (2001) Effects of lifestyle and work-related physical activity on the degree of lumbar lordosis and chronic low back pain in a Middle East population. J Spinal Disord 14:283-292

25. Picavet HS, Schuit AJ (2003) Physical inactivity: a risk factor for low back pain in the general population? J Epidemiol Community Health 57:517-518. doi: 10.1136/jech.57.7.517

26. Power C, Frank J, Hertzman C, et al. (2001) Predictors of Low Back Pain Onset in a Prospective British Study. Am J Public Health 91:1671-1678

27. Sandler RD, Sui X, Church TS, et al. (2014) Are flexibility and muscle-strengthening activities associated with a higher risk of developing low back pain? J Sci Med Sport 17:361-365. doi: 10.1016/j.jsams.2013.07.016

28. Shiri R, Solovieva S, Husgafvel-Pursiainen K, et al. (2013) The role of obesity and physical activity in non-specific and radiating low back pain: the Young Finns study. Semin Arthritis Rheum 42:640-650. doi: 10.1016/j.semarthrit.2012.09.002

29. Sitthipornvorakul E, Janwantanakul P, Lohsoonthorn V (2015) The effect of daily walking steps on preventing neck and low back pain in sedentary workers: a 1-year prospective cohort study. Eur Spine J 24:417-424. doi: 10.1007/s00586-014-3577-3

30. Taanila HP, Suni JH, Pihlajamaki HK, et al. (2012) Predictors of low back pain in physically active conscripts with special emphasis on muscular fitness. Spine J 12:737-748. doi: 10.1016/j.spinee.2012.01.006

31. Thiese MS, Hegmann KT, Garg A, et al. (2011) The predictive relationship of physical activity on the incidence of low back pain in an occupational cohort. J Occup Environ Med 53:364-371. doi: 10.1097/JOM.0b013e31820d1633

32. Thomas E, Silman AJ, Croft PR, et al. (1999) Predicting who develops chronic low back pain in primary care: a prospective study. BMJ 318:1662-1667

33. van Oostrom SH, Monique Verschuren WM, de Vet HC, et al. (2011) Ten year course of low back pain in an adult population-based cohort--the Doetinchem cohort study. Eur J Pain 15:993-998. doi: 10.1016/j.ejpain.2011.02.007

34. Yip VY (2004) New low back pain in nurses: work activities, work stress and sedentary lifestyle. J Adv Nurs 46:430-440. doi: 10.1111/j.1365-2648.2004.03009.x

35. Zanuto EA, Codogno JS, Christofaro DG, et al. (2015) Prevalence of low back pain and associated factors in adults from a middle-size Brazilian city. Cien Saude Colet 20:1575-1582. doi: <https://dx.doi.org/10.1590/1413-81232015205.02162014>
